# SDF-1 and NOTCH signaling in myogenic cell differentiation: the role of miRNA10a, 425, and 5100

**DOI:** 10.1186/s13287-023-03429-x

**Published:** 2023-08-15

**Authors:** Bartosz Mierzejewski, Iwona Grabowska, Zuzanna Michalska, Kamila Zdunczyk, Franciszek Zareba, Aliksandra Irhashava, Marta Chrzaszcz, Magdalena Patrycy, Wladyslawa Streminska, Katarzyna Janczyk-Ilach, Marta Koblowska, Roksana Iwanicka-Nowicka, Agnieszka Gromadka, Kamil Kowalski, Maria Anna Ciemerych, Edyta Brzoska

**Affiliations:** 1 Department of Cytology, Faculty of Biology, University of Warsaw, Miecznikowa 1 St, 02-096 Warsaw, Poland; 2 Laboratory of Systems Biology, Faculty of Biology, University of Warsaw, 02-096 Warsaw, Poland; 3 Laboratory of Microarray Analysis, Institute of Biochemistry and Biophysics, Polish Academy of Sciences, 02-106 Warsaw, Poland; 4 Department of Bioinformatics, Institute of Biochemistry and Biophysics, Polish Academy of Sciences, 02-106 Warsaw, Poland

**Keywords:** Mouse, Skeletal muscle regeneration, Differentiation, Interstitial cells, Satellite cells, miRNA10a, miR151, miRNA425, miRNA5010

## Abstract

**Background:**

Skeletal muscle regeneration is a complex process regulated by many cytokines and growth factors. Among the important signaling pathways regulating the myogenic cell identity are these involving SDF-1 and NOTCH. SDF-1 participates in cell mobilization and acts as an important chemoattractant. NOTCH, on the other hand, controls cell activation and myogenic determination of satellite cells. Knowledge about the interaction between SDF-1 and NOTCH signaling is limited.

**Methods:**

We analyzed two populations of myogenic cells isolated from mouse skeletal muscle, that is, myoblasts derived from satellite cells (SCs) and muscle interstitial progenitor cells (MIPCs). First, microRNA level changes in response to SDF-1 treatment were analyzed with next-generation sequencing (NGS). Second, myogenic cells, i.e., SC-derived myoblasts and MIPCs were transfected with miRNA mimics, selected on the basis of NGS results, or their inhibitors. Transcriptional changes, as well as proliferation, migration, and differentiation abilities of SC-derived myoblasts and MIPCs, were analyzed in vitro*.* Naive myogenic potential was assessed in vivo, using subcutaneous engrafts and analysis of cell contribution to regeneration of the skeletal muscles.

**Results:**

SDF-1 treatment led to down-regulation of miR10a, miR151, miR425, and miR5100 in myoblasts. Interestingly, miR10a, miR425, and miR5100 regulated the expression of factors involved in the NOTCH signaling pathway, including *Dll1*, *Jag2,* and NICD. Furthermore, miR10a, miR425, and miR5100 down-regulated the expression of factors involved in cell migration: *Acta1*, *MMP12*, and FAK, myogenic differentiation: *Pax7*, *Myf5*, *Myod*, *Mef2c*, *Myog*, *Musk*, and *Myh3.* However, these changes did not significantly affect myogenic cell migration or fusion either in vitro or in vivo*,* except when miR425 was overexpressed, or miR5100 inhibitor was used. These two molecules increased the fusion of MIPCs and myoblasts, respectively. Furthermore, miR425-transfected MIPC transplantation into injured skeletal muscle resulted in more efficient regeneration, compared to control cell transplantation. However, skeletal muscles that were injected with miR10a transfected myoblasts regenerated less efficiently.

**Conclusions:**

SDF-1 down-regulates miR10a, miR425, and miR5100, what could affect NOTCH signaling, differentiation of myogenic cells, and their participation in skeletal muscle regeneration.

**Supplementary Information:**

The online version contains supplementary material available at 10.1186/s13287-023-03429-x.

## Introduction

Skeletal muscle regeneration is a multistep process that allows the reconstruction of skeletal muscle tissue after injury. Such damage, caused by exercise, mechanical injury or induced under experimental conditions, e.g., freezing or cardiotoxin (ctx) injection, leads to a cascade of events resulting in tissue reconstruction [1, 2]. Muscle repair covers two main phases: degeneration and regeneration. Their course is similar in response to different types of injury and also in various species analyzed [3]. Degeneration involves a loss of stability of the sarcolemma, alteration of the shape and size of muscle fibers, release of calcium ions that activate cytoskeleton-degrading calpains, and release of cytokines and growth factors both from damaged fibers and extracellular matrix (ECM). The factors mentioned above lead to the activation of tissue resident immune cells and act as chemoattractants for cells circulating in blood [4–6]. Second regenerative phase covers damaged tissue reconstruction. Factors, such as fibroblast growth factor (FGF), hepatocyte growth factor (HGF), produced by immune, endothelial, or interstitial cells (like fibroblasts) or released from ECM, activate satellite cells (SCs), i.e., muscle-specific stem cells. SCs start to proliferate, differentiate into myoblasts, and further fuse to create myotubes which finally mature into myofibers [7–9]. Eventually, other elements of the skeletal muscles are restored, including the ECM, the microvasculature, and neuromuscular junctions (NMJs) securing proper innervation [10].

Reconstruction of skeletal muscle tissue occurs properly due to the presence of SCs. These cells are located between the sarcolemma and the basal lamina of muscle fibers and are characterized by the presence of the PAX7 transcription factor. When activated, SCs proliferate and follow the myogenic program driven by proteins from the myogenic regulatory factors (MRF) family. MRFs include MYF5, MYOD, myogenin, and MRF4, which sequential expression results in terminal myogenic differentiation of SCs, i.e., myofiber formation [11, 12]. SCs are well known to be crucial for proper skeletal muscle reconstruction and their depletion results in inefficient muscle regeneration [13]. Although there are many other cell types that support the reconstruction of skeletal muscle tissue. Among them are endothelial cells, interstitial cells, or immune cells, that have been previously mentioned that act indirectly by releasing cytokines and growth factors, such as insulin-like growth factor 1 (IGF-1), transforming growth factor β (TGF-β), interleukin 6 (IL-6), IL-10, vascular endothelial growth factor (VEGF), HGF, or FGF that impact SC activation, proliferation, differentiation into myoblasts, and fusion [12, 14]. Moreover, some cell types, especially pericytes and mesoangioblasts, can contribute to tissue regeneration directly by differentiation into myoblasts and the formation of new myofibers [15, 16]. Pericytes are not well defined, but their markers include NG2, ALP, and PDGFRβ. Another marker used for the characterization of pericytes is CD146, which was recently described by Sacchetti and coworkers as the so-called MSC marker in human tissues, and by us in mouse skeletal muscles as the marker of the so-called muscle interstitial progenitor cells (MIPCs) [17–20]. Both human 'MSCs' and mouse MIPCs exhibited characteristics of pericytes. These cells were perivascularly located in the interstitial space of human and mouse skeletal muscles. Furthermore, mouse MIPCs expressed some pericyte markers, such as nestin or NG2. Finally, both human 'MSCs' and mouse MIPCs were clonogenic and myogenic, but not osteo- or chondrogenic [18, 19, 21].

One of the factors that promote cell mobilization, migration, and participation in skeletal regeneration is a stromal cell-derived factor 1 (SDF-1/CXCL12). SDF-1 is a cytokine that binds to the CXCR4 or CXCR7 receptors. The SDF-1/CXCR4 axis leads to activation of the PI3K/AKT, phospholipase C, and ERK1/2 MAPK pathways. Alternatively, SDF-1/CXCR7 acts through β-arrestin and induces the JAK/STAT, ERK1/2 MAPK or AKT pathways. We showed previously that SDF-1 increases MMP activity and expression of adhesion proteins, such as CD9, and altered actin organization via FAK (focal adhesion kinase), CDC42 (cell division control protein 42), and RAC-1 (Ras-related C3 Botulinum Toxin Substrate 1) [22–26]. We also documented that SDF-1 improves the migration of mouse myoblasts and mouse embryonic stem cells through the CXCR4 receptors. Furthermore, cells treated with SDF-1 participated more efficiently in mouse skeletal muscle regeneration [23–25]. Thus, many lines of evidence connect SDF-1 to various cellular processes; however, the knowledge about its interaction with microRNAs (miRNAs) is limited.

miRNAs are short noncoding RNAs that have the ability to post-transcriptionally regulate gene expression by binding to 3 'UTR' of target mRNAs. Such binding leads to destabilization or degradation of the transcript, which results in inhibition of its translation [27–29]. SDF-1 treatment was shown to induce the expression of many miRNAs, including let-7f-5p and miR146 in human bone marrow stromal cells [30, 31]. Let-7f was shown to stimulate the migration of human bone marrow-derived stromal cells by promoting MMP9 secretion and ECM proteolysis. Furthermore, the release of let-7f encapsulated in exosomes inhibited the growth of a breast cancer [31]. miR146 was shown to attenuate SDF-1-induced cartilage degradation in human osteoarthritis (OA) chondrocytes. The same study identified miR126 as differentially expressed in chondrocytes from OA patients. miR126 is also enriched in endothelial cells and was shown to directly down-regulate SDF-1 expression and through this migration of Sca1+/Lin1- cells in ischemia [32]. Furthermore, miR141 negatively modulates SDF-1 and is involved in an age-dependent decrease in SDF-1 levels in the bone marrow niche [33]. Finally, SDF-1 is an important regulator of tumor growth and invasion. miR454 was shown to directly target and down-regulate SDF-1 in cancer cells. Overexpression of this miRNA resulted in inhibition of pancreatic ducal adenocarcinoma cell (PDAC) growth, while miR454-depleted PDAC cells grew much faster [34].

The aim of present study was to identify microRNA involved in SDF-1 signaling. As a result, we show that SDF-1 treatment of myoblasts led to down-regulation of miR10a, miR151, miR425, and miR5100. Down-regulation of CXCR4, that is, the SDF-1 receptor, on the other hand, caused up-regulation of miR10a, miR151, miR425, and miR5100 in myoblasts. Interestingly, microarray analysis showed that three of these miRNAs, i.e., miR10a, miR425, and miR5100, regulated the level of transcripts involved in NOTCH signaling. Thus, we suggest that these molecules are involved in combing SDF-1 and NOTCH signaling in myogenic cells. For this reason, to follow the impact of these miRNAs on myogenic identity, mouse SC-derived myoblasts and MIPCs were transfected with miRNA mimics encoding abovementioned miRNAs and their inhibitors, and analyzed in vitro and in vivo. We confirmed that miR10a, miR425, and miR5100 regulated the transcripts involved in NOTCH signaling, as well as migration and myogenic differentiation. Thus, SDF-1 acting through miR10a, miR425, and miR5100 affects NOTCH signaling, migration, and differentiation of myogenic cells.

## Methods

### Animal studies

Animal studies were approved by the Warsaw Local Ethics Committee No. 1 (permit number 668/2018 and 1186/2021). All details such as animal care and monitoring or experimental procedures were included in the permit. In animal experiments (subcutaneous heterotopic transplantation, muscle injury and cell engraftment) in each group (control or treated), the experimental unit was one animal. In each case, three independent experiments were performed. Total number of animals was 64. The 2–3-month-old male C57/BL6 mice were used. No additional criteria for including or excluding animals were used. The study was randomized.

### Satellite cell (SC) isolation and culture

Satellite cells (SCs) were isolated from *Gastrocnemius*, *EDL,* and *Soleus* muscles of 2–3-month-old male C57/BL6 or C57/BL6 mice carrying the LacZ transgene at the ROSA26 locus, according to Rosenblatt et al. [35]. Briefly, mice were sacrificed by cervical dislocation, muscles were isolated, cut into smaller pieces, and incubated in 0.2% collagenase type I (Sigma-Aldrich) in Dulbecco’s modified Eagle medium (DMEM; Thermo Fisher Scientific) at 37 °C for 2 h. Single fibers were collected using pipette tips, purified twice in DMEM containing glucose 1 g/l and supplemented with 10% horse serum (HS, Thermo Fisher Scientific), 1% penicillin/streptomycin (Thermo Fisher Scientific), and 0.5% chicken embryo extract (CEE, Thermo Fisher Scientific). The suspension of muscle fibers was passed through the 21G syringe needle and filtered through the 40 µm strainer. The obtained SCs were placed directly in culture dishes coated with Matrigel® Growth Factor Reduced (GFR) basement membrane matrix (Corning) or culture plates containing cover slides coated with Matrigel® GFR Basement Membrane Matrix. Cells were expanded in DMEM with glucose 1 g/l supplemented with 10% HS, 20% FBS, 1% penicillin/streptomycin and 0.5% CEE and cultured under standard conditions, that is, 37 °C, 5% CO2. The medium was replaced every 2 days. Under such conditions, SCs started myogenic differentiation and formed SC-derived myoblasts.

### Isolation, sorting, and culture of muscle interstitial progenitor cells (MIPCs)

Muscle interstitial progenitor cells (MIPCs) were obtained from *Gastrocnemius*, *EDL,* and *Soleus* muscles of 2–3-month-old C57/BL6 or C57/BL6 male mice carrying the LacZ transgene at the ROSA26 locus, as described previously [21]. Briefly, mice were sacrificed by cervical dislocation, muscles were isolated, cut into smaller pieces, and incubated in 0.2% collagenase type I in DMEM at 37 °C for 2 h. The digested muscles were mechanically fragmented and incubated in dispase (2 U/ml) in DMEM. After digestion, a homogeneous suspension of cells was centrifuged. Cells expressing CD146 were selected using magnetic columns (MACS; Miltenyi Biotec) and antibody against CD146 conjugated with ferromagnetic particles, according to the manufacturer's protocol (Miltenyi Biotec). Finally, CD146+ cells were suspended in DMEM containing glucose 4.5 g/l, supplemented with 15% FBS and 1% penicillin/streptomycin and seeded in culture plates or culture plates containing cover slides coated with 3% gelatin (Sigma-Aldrich) in water. Cells were expanded under standard conditions: 37 °C, 5% CO2. The medium was replaced every 2 days.

### Cell transfection, SDF-1 treatment, and CXCR4 silencing

MIPCs or SC-derived myoblasts were cultured in growth medium until at least 50% confluence was reached. One hour before transfection, the medium was changed to OptiMem (Thermo Fisher Scientific) supplemented with 15% FBS. SC-derived myoblasts were transfected with predesigned Silencer Select siRNA (Life Technologies) complementary to CXCR4 encoding *mRNA (ID: s64091).* Appropriate negative control siRNA was used, according to the manufacturer's recommendation. The siRNA duplexes were diluted in DMEM to a concentration of 100 pmol, and Lipofectamine RNAiMAX (Life Technologies) was added according to the manufacturer's instructions. Mouse recombinant SDF-1 (10 ng/µl) was added to cell culture 24 h after transfection. The cells were then collected and processed 48 h after SDF-1 treatment for RNA isolation and RNA sequencing.

MIPCs and SC-derived myoblasts were transfected using Lipofectamine 3000 (Thermo Fisher Scientific) and 50 nM *mir*Vana miRNA mimics or miRNA inhibitors: miR1 or one of the following: miR10a, miR151, miR425, and miR5100 (MC10617, MC12998, MC22449, MC10515 Ambion), according to the manufacturer's protocol. Cells transfected with the miR1 mimic were considered positive controls and cells transfected with the negative control #1 mimic (4464058, Ambion) were considered negative controls. Furthermore, cells were transfected with miRIDIAN miRNA mimic transfection control conjugated with Dy547 fluorescent dye (CP-004500-01-20, Dharmacon) to evaluate transfection efficiency. The transfection procedure lasted for 48 h, and then cells were washed with PBS and further cultured in medium that supports myogenic differentiation, that is, DMEM supplemented with 2% horse serum (HS; Thermo Fisher Scientific) for 5 days. Alternatively, after 48 h of transfection, cells were suspended in Matrigel® and transplanted subcutaneously, or suspended in PBS and transplanted into the injured *Gastrocnemius* muscle.

### Cell proliferation assay

SC-derived myoblasts and MIPCs, control or transfected with mimic miRNAs, were incubated in 0.5 µM carboxyfluorescein succinimidyl ester (CFSE, Thermo Fisher) in PBS at 37 °C for 10 min. Cells were rinsed in PBS and cultured for 24 h or 48 h in growth medium under standard conditions. Cells were then rinsed in PBS, trypsinized, and subjected to flow cytometry analysis (CytoFLEX, Beckman Coulter) using CytExpert software. Unlabeled cells (negative control) and cells analyzed immediately after CFSE labeling (positive control) were included in each experiment. Three independent experiments were performed.

### Fusion index

SC-derived myoblasts and MIPCs were cultured and transfected as described above. After 48 h of transfection, cells were cultured in medium that supports myogenic differentiation for another 5 days. Finally, cells were fixed with 3% PFA in PBS for further analysis. The fusion index was calculated as a percentage of cell nuclei present within the myotubes compared to all visible nuclei. Three independent experiments were performed; nuclei were counted from 10 random fields of view.

### Migration assay—scratch assay

Migration of SC-derived myoblasts and MIPCs, control ones or transfected with mimic miRNAs, was analyzed using a scratch wound healing assay. Cells were scratched off the plate using a plastic tip to create the 'wound'. The wound healing manifested by the ability of cells to fill the created gap was observed. Pictures of the 'wound' area were taken at three time points: 0 h, 6 h, and after 24 h. The scratch area was calculated using ImageJ. Three independent experiments were performed.

### Subcutaneous heterotopic transplantation

5 × 10^5^ of control or transfected MIPCs or SC-derived myoblasts were suspended in 0.5 ml of Matrigel® GFR High Concentration (HC) basement membrane matrix (Corning). Next, aliquots of approximately 0.4 ml of Matrigel® containing cells were injected into the subcutaneous connective tissue on the back of C57/BL6 male mice. After 21 days, the transplants were isolated for further analysis. Three independent experiments were performed for each of the cell populations examined.

### Muscle injury and cell engraftment

Mice were anesthetized with isoflurane (2–3% isoflurane, 0.9 L/min oxygen). *Gastrocnemius* muscles in the hind limb were injected with 50 µl of Naja mossambica cardiotoxin (CTX) (10 µM in PBS, Sigma-Aldrich), while the collateral leg was injected with 0.9% NaCl. After 24 h from injury, ~ 5 × 10^5^ of control or transfected MIPCs and SC-derived myoblasts carrying the LacZ transgene at the ROSA26 locus were suspended in PBS and transplanted into damaged *Gastrocnemius* muscle. After 14 days of regeneration, mice were sacrificed by cervical dislocation, muscles were isolated from both legs, from tendon to tendon, and frozen in isopentane cooled with liquid nitrogen. Muscles were sectioned using cryostat (Microm HM 505N; Microm International GmbH) to obtain 7 μm thick cross sections which were then air dried, fixed with 3% PFA in phosphate buffered saline (PBS), and stored for further analyzes. Other samples were ground using mortar and pestle cooled with liquid nitrogen, and the whole protein was isolated with RIPA buffer (Thermo Fisher Scientific) supplemented with protease inhibitors (cOmplete; Sigma-Aldrich) and phosphatase inhibitors (PhosSTOP; Sigma-Aldrich).

### MicroRNA sequencing

RNA was isolated from SC-derived myoblasts transfected with siRNA complementary to mRNA encoding CXCR4 or treated with SDF-1. A total of three independent samples were collected. RNA was extracted in AROS Applied Biotechnology A/S Science Park Skejby (Denmark) with the QIAsymphony RNA extraction kit from QIAGEN (Germany) using the QIAsymphony SP Biorobot from QIAGEN (miRNA CT 400 program). MiRNA was quantified using a Qubit fluorometer, and approximately 150 bp-long barcode libraries were created. The quality of the libraries was validated using the Bioanalyzer 2100 DNA High Sensitivity Kit (Agilent, USA). Libraries' concentrations were measured with the KAPA Library Quantification Kit during qPCR. Libraries were pooled with a 30% increase in control libraries PhiX (Illumina) and hybridized to a sequencing flow cell. Single read 50 bp sequencing was performed using an Illumina HiSeq sequencer with the use of a TruSeq rapid SBS kit (Illumina, USA). The quality assessment of the raw reads was performed with the FastQC tool. First, only reads containing adapters were selected for further analysis. Later, adapters and low-quality reads were discarded. Then, reads with identical sequences were grouped/collapsed into one entry and consequently were converted into read-count format. The miRNA expression profile was performed using Partek Flow v6.0 and Partek Genomics Suite v6.6 software (Partek Inc., St. Louis, MO, USA). Briefly, reads were aligned with the mouse reference genome (assembly mm10) using Bowtie1. Next, miRBase (release 21) was used to provide annotations for RNA fragments assigned to the mouse genome. Finally, TMM normalized mapped reads were used to infer lists of differentially expressed miRNAs between analyzed groups. Unsupervised hierarchical clustering was performed with the use of the Partek Genomics Suite on normalized reads to visualize differentially expressed miRNAs. The p-value cut-off point for differentially expressed miRNAs between groups was established at the *p* value ≤ 0.05.

### Microarray assay

Total RNA along with the microRNA fraction was extracted from control and transfected SC-derived myoblasts and MIPCs using the RNAqueous Micro Total Isolation Kit (Thermo Fisher Scientific) according to the manufacturer’s protocol. Furthermore, the integrity of the obtained RNA was analyzed with the 2100 Bioanalyzer (Agilent Technologies) using the RNA 6000 Nano Lab Chip Kit (Agilent Technologies). All RNA samples had an integrity number greater than 8.5. Whole transcriptome analysis was performed using the Clariom S Pico Assay (Thermo Fisher Scientific, Waltham, MA, USA) according to the manufacturing protocol. The prepared samples were hybridized with a single Clariom S mouse array and incubated for 16 h in the Affymetrix GeneChip Hybridization Oven 645 at 45 °C, 60 rpm. The arrays were stained with an Affymetrix GeneChip Fluidics Station 450, according to the specific fluidics protocol, and scanned with an Affymetrix GeneChip Scanner 3000 7G. The raw intensity CEL files generated by GeneChipTM Command ConsoleTM were imported into Transcriptome Analysis Console (TAC) 4.0 (Applied Biosystems). Microarray data were normalized and analyzed using Transcriptome Analysis Console 4.0 following the TAC user guide. Each variance analysis was performed by one-way ANOVA. To determine the significance of differentially expressed genes (DEGs), a cut-off point was applied for the fold change value ± 1.5 and the *p* value < 0.05 was applied. The list of detected differentially expressed transcripts was analyzed using Ingenuity Pathway Analysis (IPA, QIAGEN Inc.) software to identify significant interactions and pathways. All the analysis and the corresponding plots were performed following the software guide, limiting the IPA database information to molecules and relationships where the information was experimentally observed. The data obtained were also analyzed with and Transcriptome Analysis Console (TAC, Applied Biosystems), miRDB [36], and TargetScan [37].

### Quantified real-time PCR and miRNA expression assay (qRT-PCR)

Total RNA, including the miRNA fraction, was extracted from the control and mimic miRNA transfected SC-derived myoblasts and MIPCs using the RNAqueous-Micro Total Isolation Kit (Thermo Fisher Scientific), according to the manufacturer's protocol. RNA-based cDNA was synthesized using the RevertAid First-Strand cDNA synthesizer kit (Thermo Fisher Scientific), according to the manufacturer's protocol, under the following conditions: 25 °C for 5 min, 42 °C for 90 min, and 70 °C for 5 min. mRNA levels were evaluated using quantitative real-time PCR analysis (qRT-PCR) with TaqMan assays (Thermo Fisher Scientific) for the following genes: *Twf1, Acta1, Ilk, Mmp12, Dll1, Jag1, Jag2, Hes1, Hey1, Pax7, Myf5, Myod, Mef2c, Myog, Musk, Myh3* (Mm00808218_g1, Mm01274281_g1, Mm00500554_m1, Mm01279269_m1, Mm00496902_m1, Mm01325629_m1, Mm01342805_m1, Mm00468865_m1, Mm01354484_m1, Mm00435125_m1, Mm00521984_m1, Mm_01340842_m1, Mm00446194_m1, Mm01346929_m1, Mm01332463_m1, Mm_00725968_s1). The average expression of hypoxanthine phosphoribosyltransferase 1 (*Hprt1*; Mm03024075_m1) and glyceraldehyde-3-phosphate dehydrogenase (*Gapdh;* Mm99999915_g1) was used as a reference for further calculations. The reaction was carried out with TaqMan Gene Expression Master Mix (Thermo Fisher Scientific) using LightCycler96 (Roche) under the following conditions: preincubation 2 min,50 °C; preincubation 10 min, 95 °C; amplification (40 cycles) 15 s, 95 °C and 1 min, 60 °C. All reactions were performed in duplicates. Expression levels were calculated using 2^−(ΔCt)^ formula based on the relative expression of the average expression of *Hprt1* and *Gapdh*. MiRNA reverse transcription was carried out with the TaqMan MiRNA reverse transcription kit (Thermo Fisher Scientific) and TaqMan miRNA assays (Thermo Fisher Scientific) under the following conditions: 30 min, 16 °C; 30 min 42 °C; 5 min, 85 °C. MiRNA levels were assessed using quantitative real-time PCR analysis (qPCR). RT primers and TaqMan probes were used for specific miRNA: miR1 (002222), miR10a (002288), miR151 (001190), miR5100 (462702_mat), miR425 (001516), U6 (001973). The average expression of noncoding U6 snRNA was used as a reference for further calculations. The reaction was carried out with TaqMan Universal Master Mix II, without UNG (Thermo Fisher Scientific) using LightCycler96 (Roche) under the following conditions: preincubation 2 min, 50 °C; preincubation 10 min, 95 °C; amplification (40 cycles) 15 s, 95 °C and 1 min, 60 °C. All reactions were performed in duplicates. Three independent experiments were performed.

### Immunocytochemistry and immunohistochemistry

Skeletal muscle frozen sections (7 µm), transplants, or SC-derived control/transfected in vitro cultured myoblasts or MIPCs were fixed with 3% PFA in PBS. Next, the samples were washed in PBS and permeabilized in 0.05% Triton X100 (Sigma-Aldrich) in PBS, washed in PBS and incubated in 0.25% glycine (Sigma-Aldrich) in PBS, followed by incubation in 3% bovine serum albumin (Sigma-Aldrich) with 2% donkey serum albumin (Sigma-Aldrich) in PBS. The samples were then incubated with primary antibodies anti-cleaved NOTCH1 (4147S, Cell Signaling), anti-skeletal myosin (M7523, Sigma-Aldrich), anti-laminin (L9393, Sigma-Aldrich), anti-β-galactosidase (ab9361, Abcam) diluted 1:100 in 3% BSA with 2% donkey serum in PBS at 4 °C overnight, followed by incubation in appropriate secondary antibodies conjugated with Alexa Fluor 488 or 594 (anti-mouse, 21203; anti-rabbit 21206; anti-rabbit 21207; anti-chicken, 11039; Thermo Fisher Scientific) diluted 1: 500 in 1.5% BSA in PBS at room temperature for 2 h. Negative controls using secondary antibodies were performed. Cell nuclei were visualized by 5-min-long incubation in Hoechst 33342 (Thermo Fisher Scientific) diluted 1:1000 in PBS. The actin cytoskeleton was visualized using phalloidin conjugated with TRITC (tetramethylrhodamine B isothiocyanate). The samples were mounted with fluorescent mounting medium (Dako Cytomation) and analyzed using the LSM 700 confocal microscope (Zeiss) and ZEN software (Zeiss). The proportion of cells expressing the examined proteins was calculated from 10 fields of view on each slide, and each experiment was performed three times.

### Histological analysis

Muscle sections were stained with Gill’s hematoxylin (Sigma-Aldrich) and eosin Y (Sigma-Aldrich) and mounted in permanent aqueous mounting agent for microscopy (Dako). Sections will be analyzed using Axio Scan Z1 (Zeiss). Newly formed myofibers with centrally located nuclei were counted from 10 fields of view on each slide, and each experiment was performed three times.

### Western blot

Proteins were isolated from control or transfected MIPCs, SC-derived myoblasts, or *Gastrocnemius* muscles, control or injected with cells, using RIPA buffer (Thermo Fisher Scientific). Twenty-five micrograms of total protein lysate were denatured by boiling in Laemmli buffer, separated using SDS-Page electrophoresis, and transferred to PVDF membranes (Roche). The membranes were blocked with 5% milk/Tris buffered saline (TBS) for 1 h and incubated with primary antibodies diluted 1:1000 in 5% milk/TBS, at 4 °C, overnight, followed by secondary antibodies diluted 1:20,000, at room temperature, for 2 h. The protein bands were then visualized with the SuperSignal West Dura Extended Duration Substrate (Thermo Scientific) and exposed to a chemiluminescence positive film (Amersham Hyperfilm ECL, GE Healthcare). The film was developed using a developer and fixer (Fuji). The density of the examined bands was compared with the density of α-tubulin bands. The following primary antibodies were used: mouse monoclonal anti-β-actin (sc-47778; Santa Cruz), rabbit monoclonal anti-NICD (4147; Cell Signaling), rabbit polyclonal anti-FAK (3285; Cell Signaling), rabbit polyclonal anti-phospho FAK (3283; Cell Signaling), mouse monoclonal anti-MYOD (554130; BD Biosciences), rabbit polyclonal anti-MCK (SAB4500267 Sigma-Aldrich), and mouse monoclonal anti-α-tubulin (T5168; Sigma-Aldrich). Secondary antibodies used: rabbit anti-mouse IgG conjugated with peroxidase (A9044; Sigma-Aldrich) and goat anti-rabbit IgG conjugated with peroxidase (A5420; Sigma-Aldrich), goat anti-rabbit IgG conjugated with peroxidase (sc-2004; Santa Cruz). Three independent experiments were performed. Blots were analyzed using Gel Doc XR+ and Image Lab 5.1 (BioRad).

### Statistical analysis

The Gaussian distribution of values was analyzed with the Shapiro–Wilk normality test. The fold change was calculated by comparing the average values of the nontreated samples to those of all samples. Data were analyzed using the t-test or the one-way ANOVA test and post hoc with Dunnett’s multiple comparisons test. All data were compared to results coming from analyzes of control group, i.e., nontreated cells. Differences were considered statistically significant when *p* < 0.05 (marked with asterisks, *p < 0.05; **p < 0.005; ***p < 0.001; ****p < 0.0001). The mean value and standard deviation were shown in each graph presented. All statistical analyses were performed using GraphPad 7 software (Prism).

## Results

### Changes in miRNA level after SDF-1 treatment or miRNA mimic transfection

**Fig. 1:**
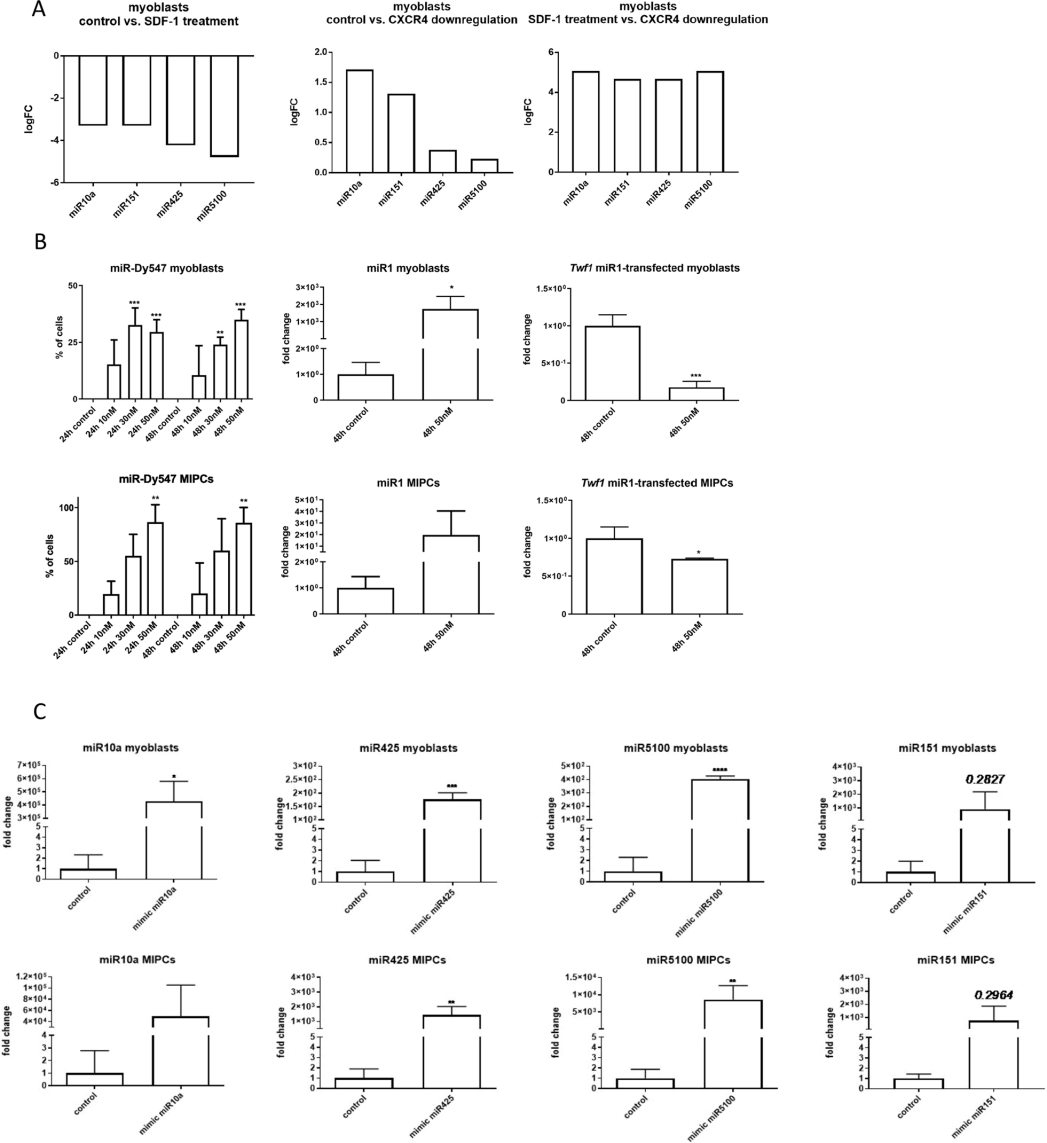
Analyzes of miRNA expression, transfection efficiency and efficacy of SC-derived myoblasts or MIPCs. **A** miR10a, miR151, miR425, and miR5100 expression in SC-derived myoblasts after SDF-1 treatment, down-regulation of CXCR4, and combined SDF-1 treatment and down-regulation of CXCR4; **B** Transfection efficiency of SC-derived myoblasts and MIPCs assessed by the transfection with 10 nM, 30 nM, or 50 nM miR-Dy547 and analyzed after 24 or 48 h. Expression level of miR1 in SC-derived myoblasts and MIPCs transfected with 50 nM miR1 mimic and analyzed 48 h after transfection. Expression of miR1 direct target—*Twf1* in control or miR1 mimic transfected SC-derived myoblasts and MIPCs analyzed 48 h after transfection; **C** Expression of miR10a, miR151, miR425, miR5100 in control or miRNA mimic transfected SC-derived myoblasts and MIPCs. Three independent analyses were performed, all reactions were performed in duplicates. Differences were considered statistically significant when *p* < 0.05 (marked with asterisks, *p < 0.05; **p < 0.005; ***p < 0.001; ****p < 0.0001)

In our previous study, we observed that SDF-1 impacts satellite cell (SC)-derived myoblast migration and their participation in skeletal muscles regeneration [23–26, 38]. Moreover, it impacts few miRNA expression. However, little is known about the nature of miRNA interactions with SDF-1 pathway. To select miRNAs involved in SDF-1 regulation, next-generation sequencing was performed using control, SDF-1 treated, and *Cxcr4* siRNA transfected SC-derived myoblasts (Fig. 1A). As a result, we selected 4 molecules which levels differed significantly between SDF-1-treated and *Cxcr4-*down-regulated myoblasts. The level of miR10a, miR151, miR425, and miR5100 increased significantly in myoblasts in which *Cxcr4* expression was down-regulated, as compared to control or SDF-1 treated cells (Fig. 1A). Therefore, we decided to analyze the effect of these miRNAs on myogenic cell migration and differentiation using miRNA mimics or miRNA inhibitors. Recently, we characterized the population of mouse interstitial progenitor cells (MIPCs), as CD146+ myogenic cells present between muscle fibers and different from satellite cells [21]. Such cells were described for the first time in human skeletal muscles by Sacchetti and coworkers [18]. At first, we optimized cell transfection conditions (Fig. 1B, C). To this point, SC-derived myoblasts and MIPCs were transfected either with 10 nM, or 30 nM, or 50 nM miRDy547, i.e., fluorescently labeled control miRNA, and the proportion of transfected cells was evaluated after 24 and 48 h (Fig. 1B). The proportion of positive cells was the highest when SC-derived myoblasts and MIPCs were transfected with 50 nM miRDy547 and analyzed 48 h later. Under such conditions 34.8% ± 4.6 of SC-derived myoblasts and 86.5% ± 14.1 of MIPCs were successfully transfected. Next, we verified whether we were able to achieve down-regulation of selected miRNA targets. To this point, we transfected SC-derived myoblasts and MIPCs with miR1 mimics (50 nM) and showed that the level of this miRNA increased significantly (Fig. 1B). Consequently, the level of the miR1 target, that is, *Twf1* (Twinfilin Actin Binding Protein 1), decreased significantly in transfected cells (Fig. 1B). After this initial analysis, SC-derived myoblasts and MIPCs were transfected with miRNA mimics encoding miR10a, miR151, miR425, and miR5100. In each case, a significant increase in miRNA level was observed in analyzed cells (Fig. 1C).

### Changes in the transcriptome after miRNA mimics and inhibitors transfection

**Fig. 2:**
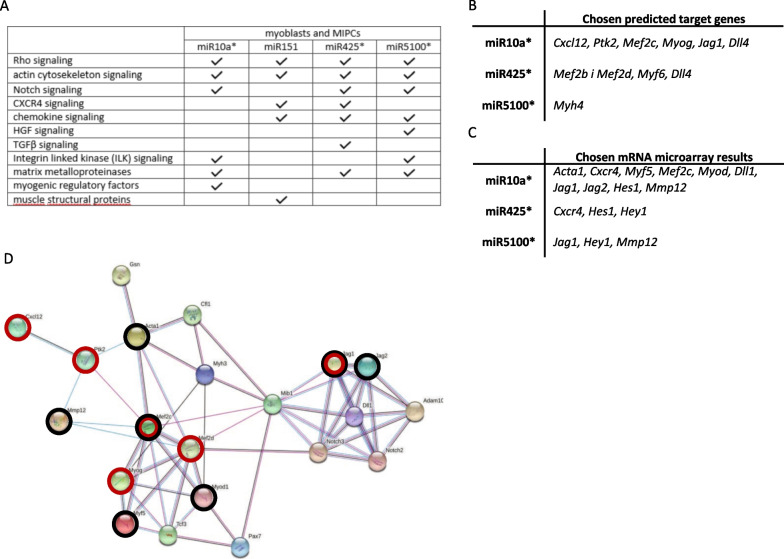
Impact of miR10a, miR151, miR425, and miR5100 mimics transfection on SC-derived myoblasts or MIPC transcriptome. **A** Signaling pathways found to be affected in SC-derived myoblasts or MIPCs transfection of miR10a, miR151, mIR425 or miR5100 using IPA and TAC; **B** Predicted miR10a, miR425 or miR5100 targets found by miRDB and TargetScan databases; **C** Statistically significant differentially expressed genes in SC-derived myoblasts and MIPC transfected with mIR10a, miR425, or miR5100 mimics; **D** STRING analysis of selected predicted targets of miR10a, miR425 or miR5100 (marked with red circles) and selected differentially expressed genes (marked with black circles) in SC-derived myoblasts and MIPC transfected with miR10a, miR425 or miR5100. Three independent analyzes were performed

The transcriptome of transfected cells was analyzed using mRNA microarrays (Fig. 2, Additional file 1: Fig. S1, S2). Transfection with miR10a, miR151, miR425, miR5100 mimics or appropriate inhibitors targeting these miRNAs changed the level of many transcripts involved in cell proliferation, adhesion, and migration (Additional file 1: Fig. S1). Bioinformatic approach using Ingenuity Pathway Analysis (IPA) and Transcriptome Analysis Console (TAC) showed that transfection with miR10a, miR151, miR425, miR5100 mimics or those miRNA inhibitors changed the level of transcripts of few canonical signaling pathways, such as, RHO, chemokine-mediated pathways, CXCR4, NOTCH, HGF, TGFβ, integrin-linked kinase (ILK), as well as those involved in the organization of the actin cytoskeleton (Fig. 2A). Furthermore, miR10a, miR425, miR5100 mimics or their inhibitors changed the level of transcripts encoding matrix metalloproteinases, myogenic regulatory factors, Ilk, and factors involved in NOTCH signaling. Since miR151 did not regulate expression of proteins involved in NOTCH signaling, miR10a, miR425, and miR5100 were chosen for further analysis. Using miRDB and TargetScan databases, we searched for predicted target genes of miR10a, miR425, and miR5100 as well as the transcripts which levels changed significantly after transfection with these miRNAs, as shown using mRNA microarrays. As the number of transcripts potentially regulated by analyzed miRNA and those selected as a result of transcriptome analyzes was large, we focused on the transcripts that could participate in the regulation of cell adhesion, migration, myogenic differentiation, and NOTCH signaling (Fig. 2B, C, Additional file 1: Fig. S2). The miRDB and TargetScan databases search showed that miR10a, miR425, and miR5100 can potentially recognize some transcripts that encode factors involved in abovementioned processes. Among such mRNAs were *Cxcl12* (encoding SDF1*)* and *Ptk2* (encoding focal adhesion kinase; FAK) involved in cell migration and adhesion, *Mef2b/c/d, Myod, Myf6* (MRF4), and *Myh4* involved in myogenic differentiation, as well as *Jag1* and *Dll4* involved in NOTCH signaling (Fig. 2B). Transcriptome analyses revealed that some of these predicted targets, such as *Mef2c* or *Jag1,* were differentially expressed in SC-derived myoblasts and MIPCs after miR10a, miR425, or miR5100 overexpression. Furthermore, the level of many other transcripts, not included in the miRDB and TargetScan databases, has also changed. Among them were *Acta1, Cxcr4,* and *Mmp12* involved in cell migration, *Myf5,* involved in myogenic differentiation, and *Dll1, Jag2, Hes1, Hey1* involved in NOTCH signaling (Fig. 2C). Next, we performed a STRING analysis which allowed us to show that the molecules listed in databases and differentially expressed in SC-derived myoblasts or MIPCs after miR10a, miR425, and miR5100 transfection formed the network of interactions (Fig. 2D).

### The influence of miRNA mimic and inhibitor transfection on transcript and protein levels

**Fig. 3:**
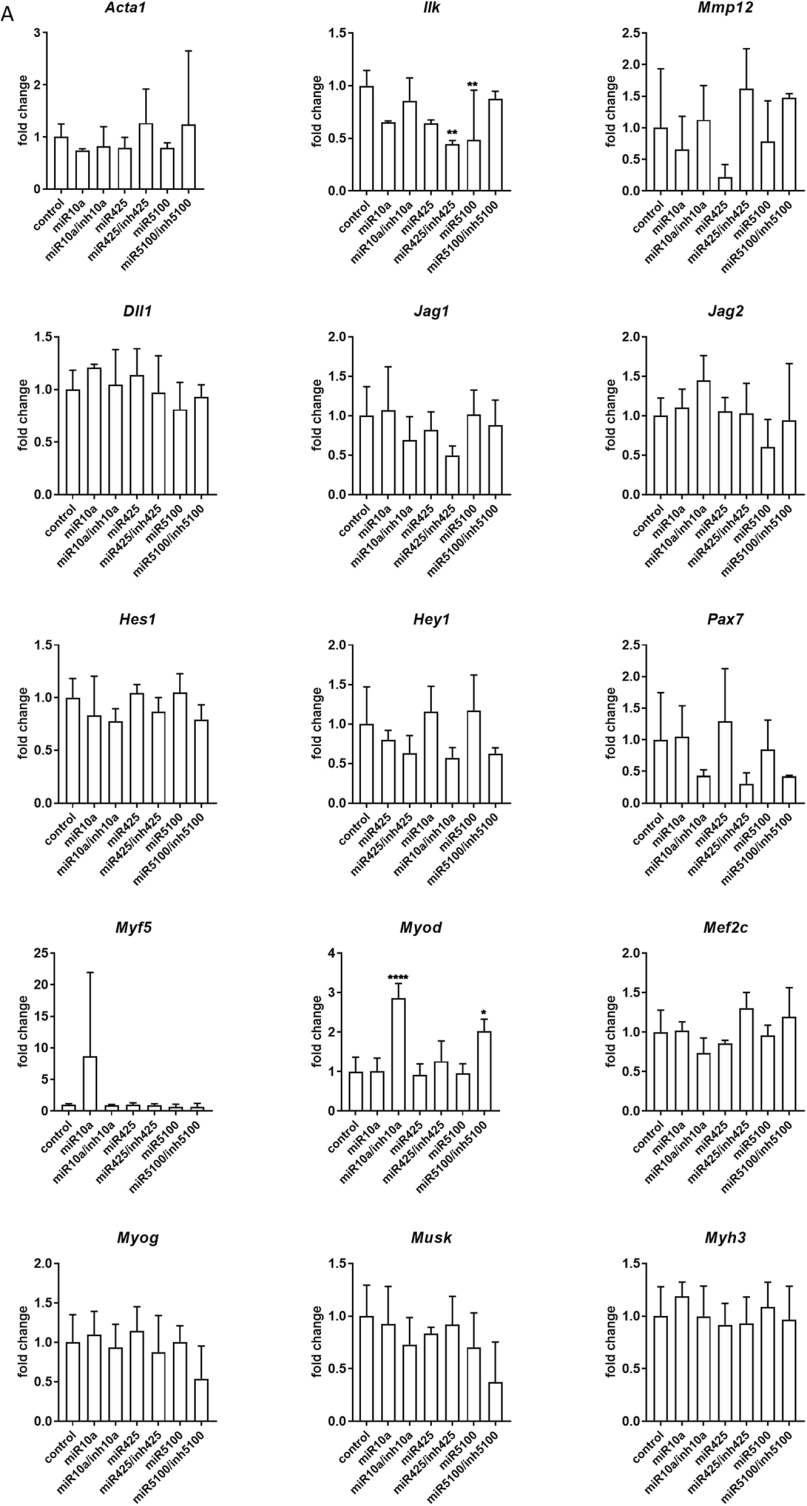

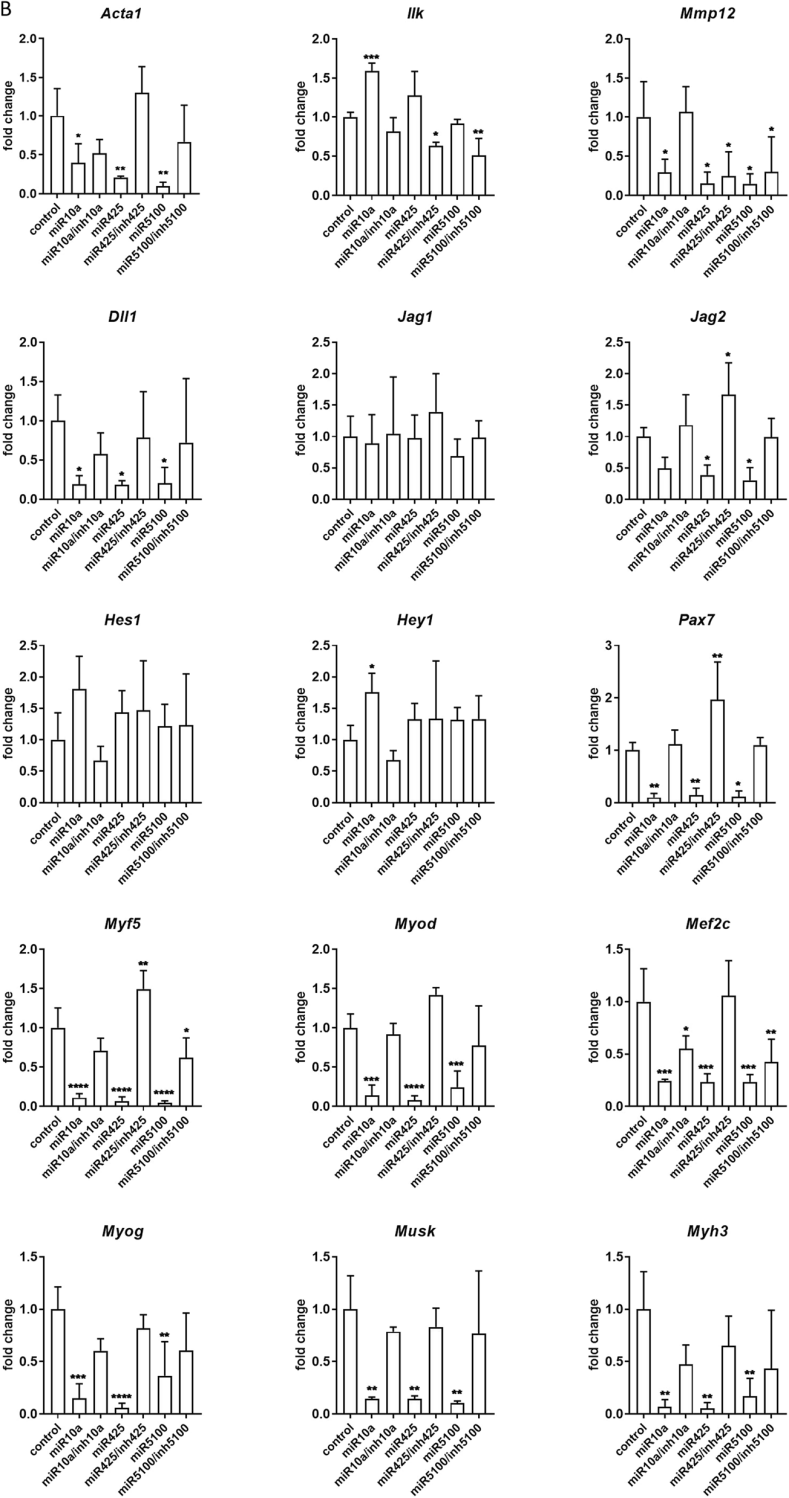

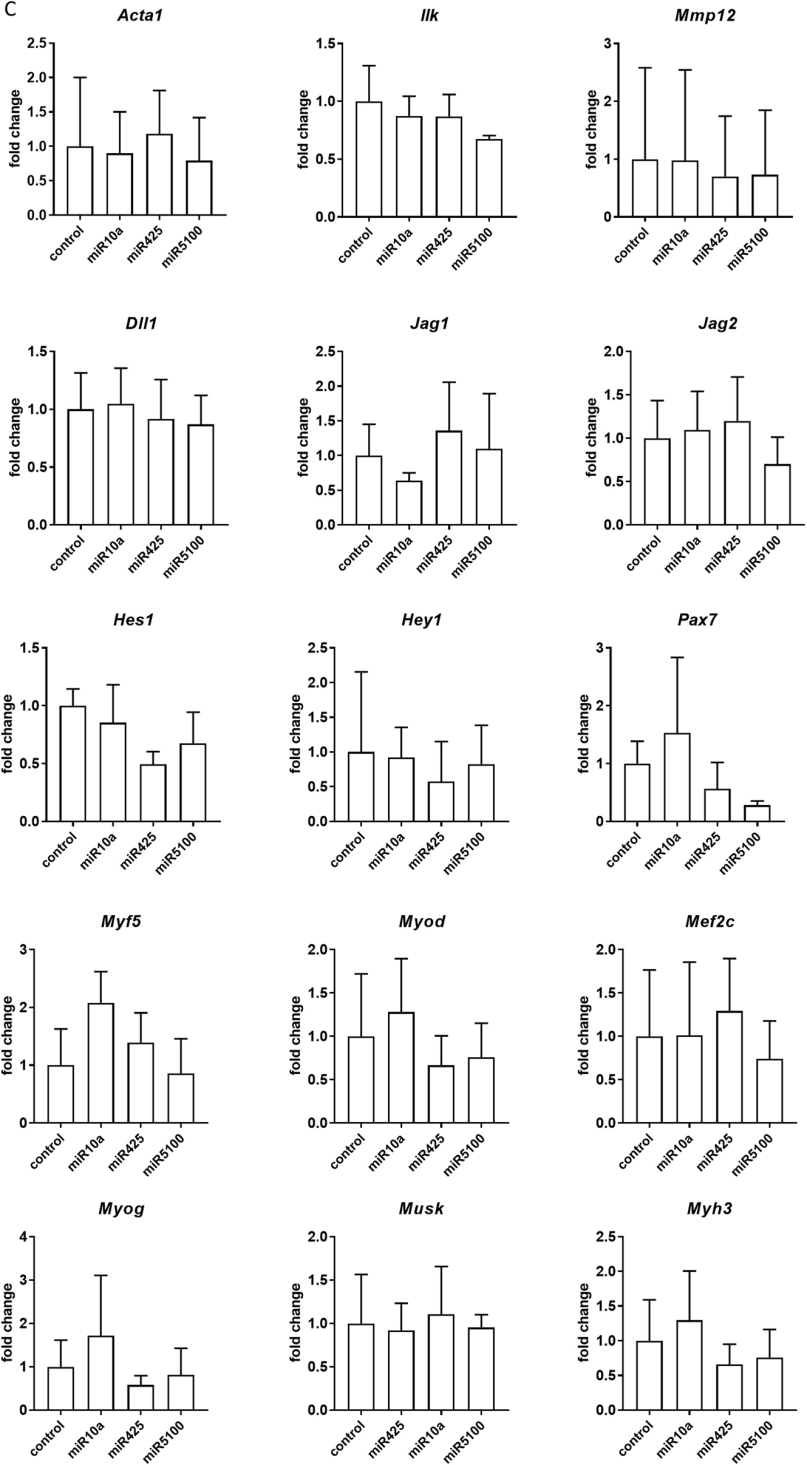

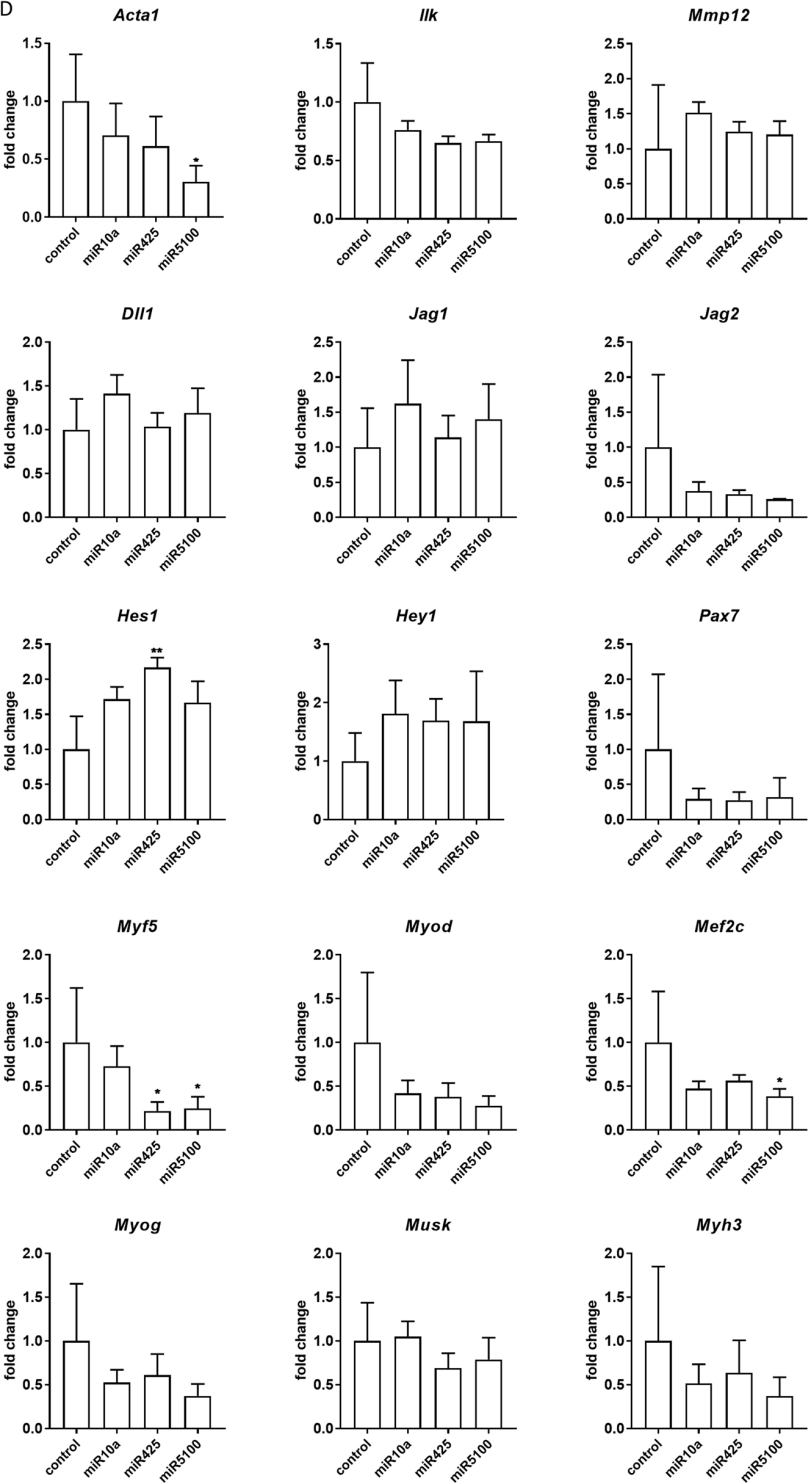
Analysis of selected transcripts in control and miR10a, miR425 or miR5100 mimics or mimics together with inhibitors miRNA transfected SC-derived myoblasts or MIPCs. **A** Control and transfected SC-derived myoblasts analyzed 48 h post transfection; **B** Control and transfected MIPCs analyzed 48 h post transfection; **C** Control and transfected SC-derived myoblasts transfected for 48 h and cultured for 5 days; **D** Control and transfected MIPCs transfected for 48 h and cultured for 5 days. Three independent analyzes were performed, all reactions were performed in duplicates. Differences were considered statistically significant when *p* < 0.05 (marked with asterisks, *p < 0.05; **p < 0.005; ***p < 0.001; ****p < 0.0001)

Changes in the selected transcript levels in cells transfected either with miR10a, or miR425, or miR5100 mimics or mimics together with miRNA inhibitors were verified by qPCR (Fig. 3, Additional file 1: Fig. S3). First, we focused on transcripts involved in cell adhesion and migration, such as skeletal muscle actin α1, (*Acta1*), integrin-linked kinase (*Ilk*), and matrix metalloproteinase 12 (*Mmp12*) (Fig. 3). 48h after, transfection, the level of *Acta1, Ilk,* and *Mmp12* did not change significantly in SC-derived myoblasts, except *Ilk,* which level decreased in miR5100 or mir425 inhibitor transfected cells (Fig. 3A). However, a significant decrease in *Acta1* and *Mmp12* expression was observed in MIPCs transfected with miR10a, miR425, and miR5100 mimics (Fig. 3B). The level of *Ilk* mRNA increased significantly in MIPCs transfected with miR10a. Importantly, the level of *Acta1*, *Mmp12,* and *Ilk* did not differ between MIPCs transfected with miRNA mimics and inhibitors compared to control cells. Then, we focused on transcripts connected with NOTCH signaling, such as *Dll1, Jag1, Jag2, Hes1,* and *Hey1*. Forty-eight hours after transfection, no significant changes in analyzed gene expression were observed in SC-derived myoblasts (Fig. 3A). A significant decrease in *Dll1* and *Jag2* was observed for miR425 and miR5100 mimic and inhibitor transfected MIPCs. A similar, but not significant, trend in *Dll1* and *Jag2* was observed for miR10a mimic transfected MIPCs. The levels of *Dll1* and *Jag2* did not change in miR10a, miR425, and miR5100 mimics and inhibitor transfected MIPCs, as compared to control cells. Only in case of miR425 mimic and inhibitor transfected MIPCs, the significant increase in *Jag2* level was noticed. The last group of transcripts analyzed was these encoding factors involved in myogenic differentiation. i.e., *Pax7, Myf5, Myod, Mef2c,* myogenin (*Myog*), muscle-specific kinase (*Musk*), and myosin heavy chain 3 (*Myh3*). After 48 h, in SC-derived myoblasts transfected with selected mimics the level of transcripts did not change (Fig. 3A). However, the *Myod* level increased in SC-derived myoblasts transfected with miR10a and miR5100 mimics and inhibitors. Furthermore, miR10a, miR425, or miR5100 mimics significantly reduced levels of *Pax7, Myf5, Myod, Mef2c, Myog,* and *Myh3* mRNA in transfected MIPCs (Fig. 3B). In MIPCs transfected with selected miRNA mimics and inhibitors, the level of *Pax7, Myf5, Myod, Mef2c, Myog,* and *Myh3* was the same as in control cells. Only in case of miR425 mimic and inhibitor transfected MIPCs the significant increase in *Pax7 and Myf5* level was noticed. Thus, we concluded that in MIPCs the levels of transcripts involved in cell adhesion and migration, such as *Acta1* and *Mmp12,* Notch signaling, i.e., *Dll1* and *Jag2, a*nd myogenic differentiation, i.e., *Pax7, Myod, Myf5, Mef2c, Myog,* and *Myh3* were regulated by miR10a, miR425, and miR5100. Next, to follow the influence of transfection on SC-derived myoblast and MIPC differentiation, these cells were transfected (48 h) and cultured for additional 5 days. No significant changes were observed in the level of the analyzed transcripts (Fig. 3B, D). Only the level of *Acta1* and *Mef2c* in miR5100 mimics transfected MIPCs, and *Myf5* in miR425 or miR5100 mimics transfected MIPCs was significantly reduced.

**Fig. 4:**
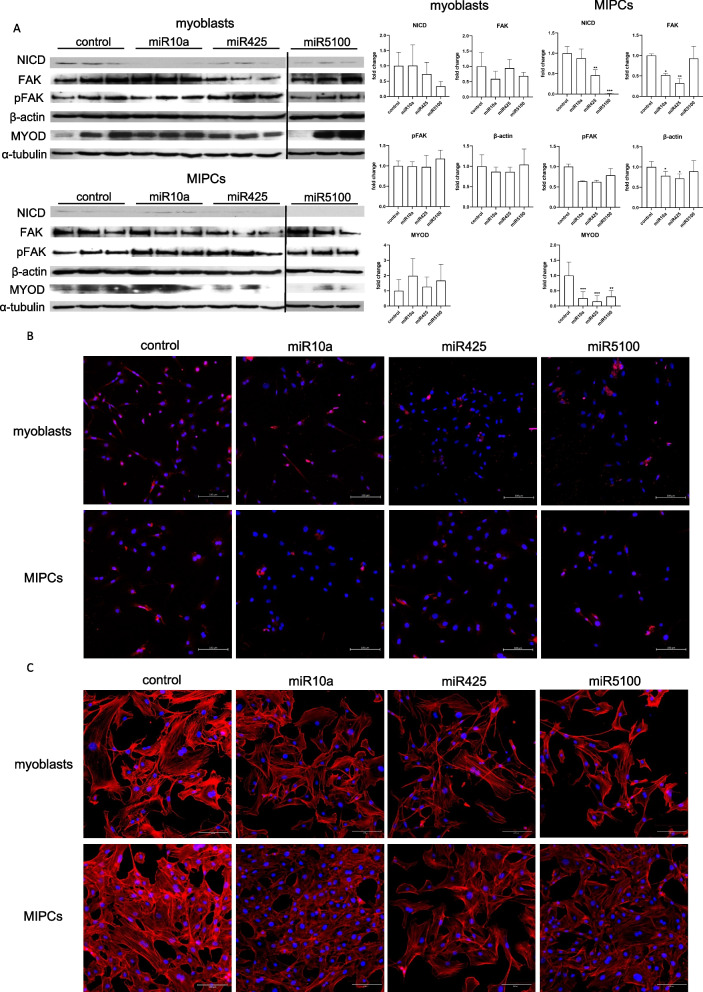
Analysis of the selected proteins in control and miR10a, miR425 or miR5100 mimics transfected SC-derived myoblasts or MIPCs. **A** Western blot analysis of NICD (~ 110 KDa), FAK (~ 120 KDa), pFAK (~ 120 KDa), β-actin (~ 40 KDa), MYOD (~ 45 KDa) and α-tubulin (~ 50 KDa). Full-length blots/gels are presented in Additional file 1: Fig. S4; **B** Immunolocalization of NICD in SC-derived myoblasts and MIPCs; NICD—red; cell nuclei—blue; **C** Immunolocalization of the actin (red) in SC-derived myoblasts and MIPCs; actin, red, cell nuclei, blue. Three independent analyzes were performed. Differences were considered statistically significant when *p* < 0.05 (marked with asterisks, *p < 0.05; **p < 0.005; ***p < 0.001; ****p < 0.0001). Scale: 150 µm

To verify our observations, we performed Western blotting and immunolocalization of selected proteins (Fig. 4, Additional file 1: Fig. S5). Western blot analysis showed a significant decrease in the NOTCH intracellular domain (NICD) in MIPCs transfected with miR425 or miR5100 mimics, compared to control cells (Fig. 4A). No such decline was observed in control or transfected SC-derived myoblasts. Immunocytochemistry confirmed that the level of cells in which NICD was localized in the cell nucleus, that is, the NOTCH pathway was active, decreased in miR425 or miR5100 mimic transfected MIPCs (Fig. 4B). Furthermore, also the level β-actin involved in the regulation of cell motility decreased in MIPCs transfected with miR10a, miR425 mimics compared to control cells (Fig. 4A). Although the organization of actin filaments was not changed in transfected SC-derived myoblasts or MIPCs, the density of actin filaments was lower in miR10a, miR425, or miR5100 mimics transfected MIPCs, compared to control, i.e., non-transfected cells (Fig. 4C). Thus, overexpression of miR425 or miR5100 mimic led to the decrease in *Dll1, Jag2*, at the mRNA level and NICD at the protein level. Furthermore, miR10a and miR425 led to drop in β-actin as well as total and activated FAK levels, that is, proteins involved in cell migration and adhesion. Furthermore, a significant decrease in MYOD was observed in miR10a, miR425, and miR5100 mimic transfected MIPCs (Fig. 4A).

### The influence of miRNA mimics transfection at SC-derived myoblasts and MIPC migration, proliferation, and fusion in vitro, after heterotopic transplantation, and during skeletal muscle reconstruction in vivo

**Fig. 5:**
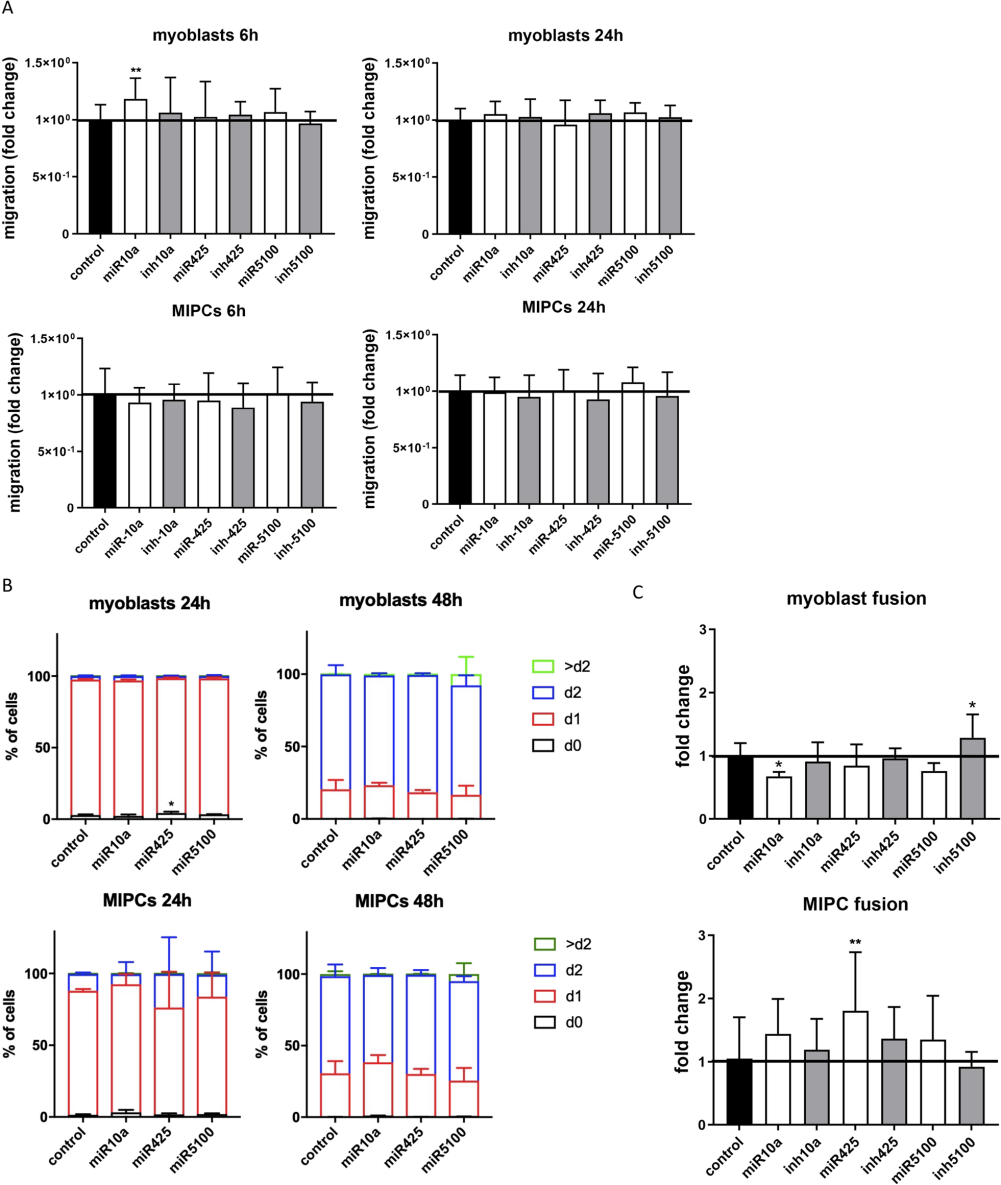
Analysis of miR10a, miR425, or miR5100 mimics or inhibitors transfection on migration, proliferation, and fusion of SC-derived myoblasts or MIPCs. **A** Migration of control or transfected SC-derived myoblasts or MIPCs analyzed 6 and 24 h after performing a scratch; **B** CFSE assay of control or transfected SC-derived myoblasts or MIPCs 24 or 48 h after adding CFSE. The proportion of cells that underwent one (d1), two (d2), or more (> d2) divisions were shown; **C** Fusion index of SC-derived myoblasts or MIPCs in control or transfected. Three independent analyzes were performed. Differences were considered statistically significant when *p* < 0.05 (marked with asterisks, *p < 0.05; **p < 0.005)

To examine whether the observed changes had an impact on cell functions, we analyzed migration, proliferation, and fusion of SC-derived myoblasts and MIPCs transfected with MiRNA mimics or inhibitors (Fig. 5). The only miRNA that increased 6 h after transfection myoblast migration was miR10a (Fig. 5A). In case of other miRNAs, they did not impact the ability of transfected SC-derived myoblasts and MIPCs to migrate, as compared to the control, even 24 h after transfection. Transfection with selected miRNAs did not significantly affect cell proliferation (Fig. 5B). Furthermore, 7 days after miR10a transfection, the fusion index of SC-derived myoblasts and MIPCs decreased significantly. It increased in the case of transfection with miR5100 inhibitor. Interestingly, MIPCs fused with each other more efficiently after transfection with miR425. Furthermore, in the case of SC-derived myoblasts or MIPCs transfected with miR151 mimic, no significant changes in proliferation and fusion were observed (Additional file 1: Fig. S4).

**Fig. 6:**
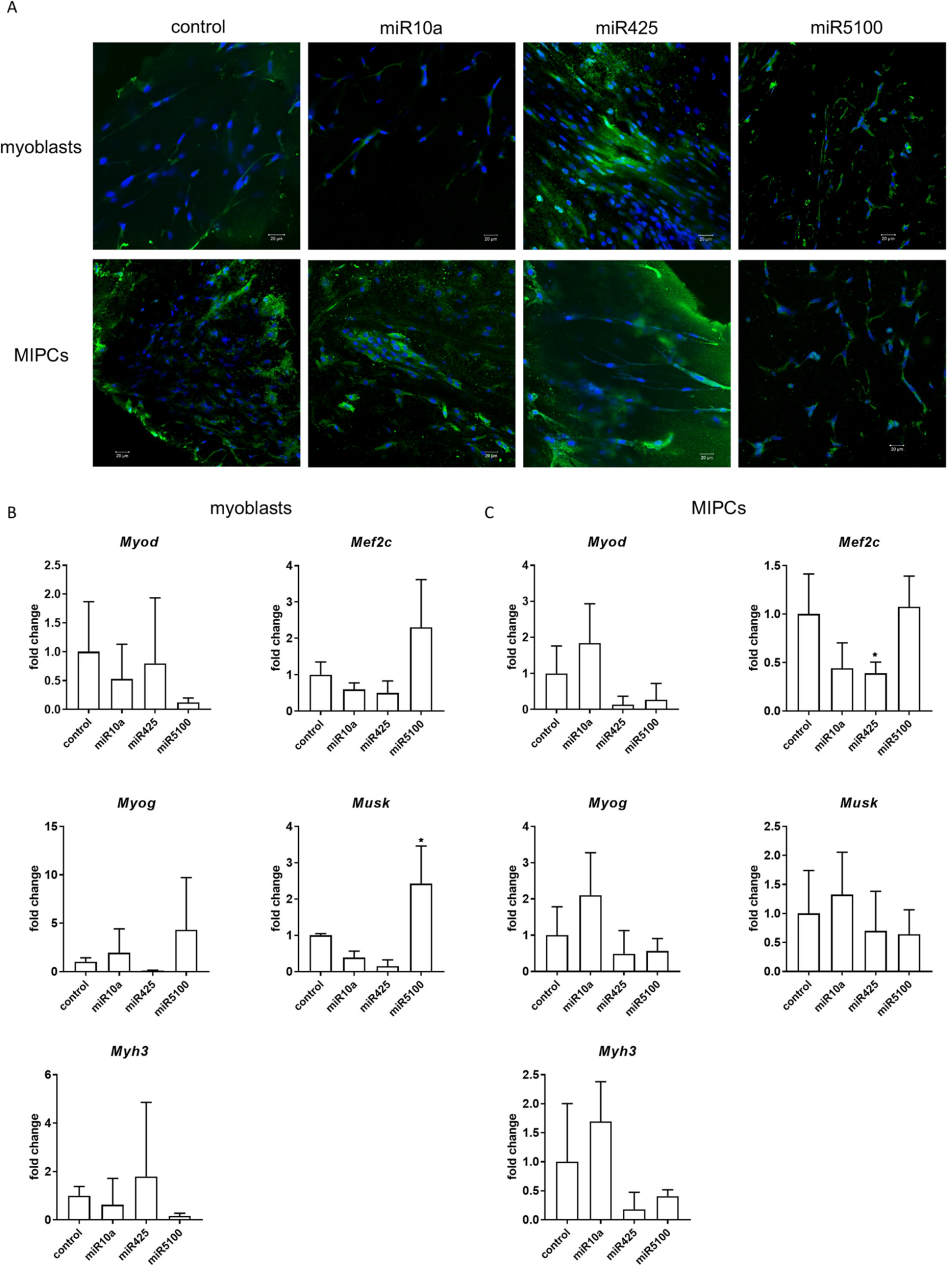
In vivo myogenic differentiation of control or miR10a, miR425 or miR5100 mimics transfected SC-derived myoblasts or MIPCs subcutaneously transplantated in Matrigel® and analyzed 21 days after transplantation. **A** Immunolocalization of skeletal muscle myosin in sections of Matrigel® containing differentiating cells; myosin—green, cell nuclei—blue; **C** Level of myogenesis associated transcripts in cells transplanted within Matrigel®. Three independent analyzes were performed, all reactions were performed in duplicates. Differences were considered statistically significant when *p* < 0.05 (marked with asterisks, *p < 0.05). Scale: 20 µm

Next, to follow cell differentiation in vivo*,* in the absence of exogenous myoblasts and factors secreted within the muscle niche, cells transfected with miR10a, miR425, or miR5100 mimics were embedded in Matrigel® and subcutaneously transplanted into mouse (Fig. 6). Matrigel® containing either differentiated SC-derived myoblasts or MIPCs was isolated and analyzed 21 days after transplantation. The immunodetection of skeletal muscle myosin documented that control SC-derived myoblasts and MIPCs followed myogenic differentiation and formed myotubes in vivo (Fig. 6A). Similarly, cells transfected with miR10a, miR425, or miR5100 were able to follow the myogenic program in vivo. However, no significant changes in *Myod, Mef2c, Myog, Musk,* and *Myh3* levels were observed in transfected cells compared to the control ones. Only in the case of MIPCs transfected with miR425, the *Mef2c* mRNA level was down-regulated (Fig. 6B, C).

**Fig. 7:**
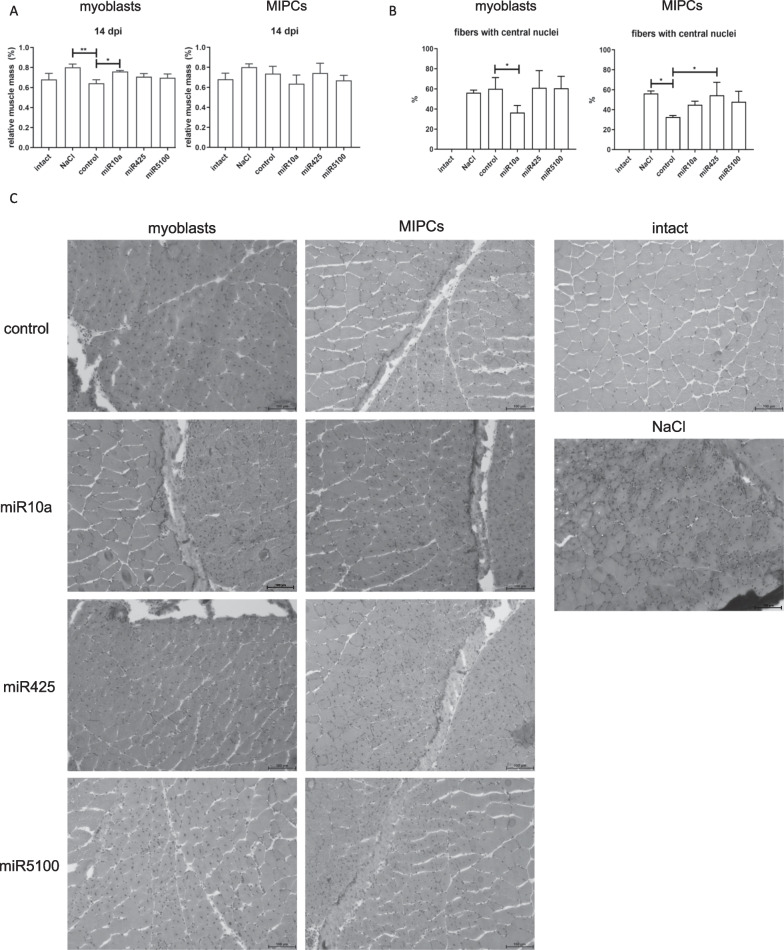
Impact of control or miR10a, miR425 or miR5100 mimics transfected SC-derived myoblasts or MIPCs at regeneration of injured skeletal muscles—muscle weight, muscle size, and histology. **A** Relative muscle mass of intact or injured *Gastrocnemius* muscle injected either with NaCl, or control or transfected SC-derived myoblasts or MIPCs analyzed at 14th day of regeneration; **B** Proportion of myofibers with centrally located nuclei in intact or injured *Gastrocnemius* muscle injected either with NaCl, or control or transfected SC-derived myoblasts or MIPCs analyzed at 14th day of regeneration; **C** Histological analysis of injected either with NaCl, or control or transfected SC-derived myoblasts or MIPCs; hematoxylin staining analyzed at 14th day of regeneration. Three independent analyzes were performed, all reactions were performed in duplicates. Scale: 100 µm

**Fig. 8:**
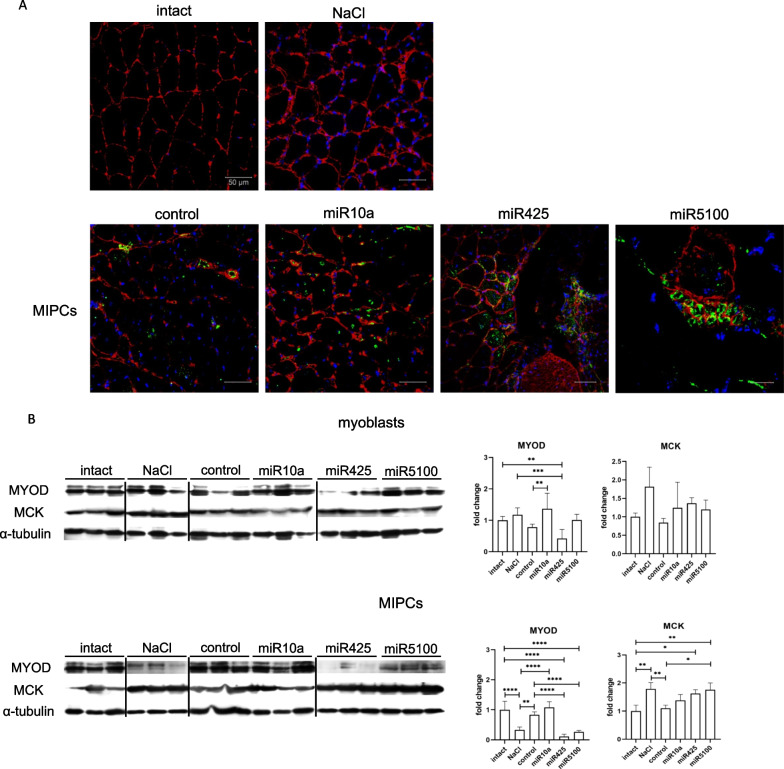
Impact of control or miR10a, miR425 or miR5100 mimics transfected SC-derived myoblasts or MIPCs at regeneration of injured skeletal muscles—localization and myogenic markers expression. **A** Immunolocalization of laminin and β-galactosidase in intact or injured *Gastrocnemius* muscle injected either with NaCl, or control or transfected MIPCs analyzed at 14th day of regeneration; laminin—red; β-galactosidase—green; cell nuclei—blue; Scale: 50 µm; **B** Western blot analysis of MYOD (~ 45 KDa), MCK (~ 45 KDa) and α-tubulin (~ 50 KDa) in intact or injured *Gastrocnemius* muscle injected either with NaCl, or control or transfected SC-derived myoblasts or MIPCs analyzed at 14th day of regeneration. Full-length blots/gels are presented in Additional file 1: Fig. S5. Three independent analyzes were performed. Differences were considered statistically significant when *p* < 0.05 (marked with asterisks, *p < 0.05; **p < 0.005; ***p < 0.001; ****p < 0.0001)

To analyze the ability of transfected cells to participate in muscle regeneration, we transplanted them into injured G*astrocnemius* muscle (Fig. 7). In this experiment, SCs and MIPCs were isolated from mice that carried lacZ transgene at the ROSA26 locus, to distinguish them from recipient tissue on the basis of β-galactosidase presence or activity of this enzyme. The injured muscle regenerated effectively as was assessed 14 days after the injury. No significant changes in muscle mass were observed between intact and NaCl injected muscle or muscles that were injected with control or transfected SC-derived myoblasts or MIPCs (Fig. 7A). Muscle mass and architecture did not differ significantly between regenerating control muscles and those receiving control or transfected SC-derived myoblasts or MIPCs (Fig. 7A). The only exception were muscles injected with miR10a transfected SC-derived myoblasts. In this case, muscle mass was higher, as compared to those injected with control cells. The proportion of new muscle fibers with centrally localized nuclei was similar in injured muscle injected with NaCl, control SC-derived myoblasts or the those transfected with mir425 or miR5100 mimics (Fig. 7B, C). The lower number of new muscle fibers with centrally located nuclei was observed in muscles injected with miR10a transfected SC-derived myoblasts. Interestingly, transplantation of control MIPCs decreased the number of new muscle fibers with centrally located nuclei, while in the case of miR425 transfected cells, the number of such fibers increased (Fig. 7B, C). Both transplanted SC-derived myoblasts and MIPCs were located between muscle fibers and participated in the formation of new myofibers (Fig. 8A). Furthermore, transplanted MIPCs were localized in blood vessel wall. The efficiency of muscle regeneration was also confirmed by Western blotting (Fig. 8B, Additional file 1: Fig. S6). The level of MYOD was higher, and the level of MCK was lower in injured muscles transplanted with control MIPCS then in those that were NaCl injected (Fig. 8B). Furthermore, the level of MYOD was lower and the level of MCK was higher in muscles transplanted with miR425 transfected MIPCs than in muscles transplanted with control ones (Fig. 8B). In addition, the same effect was observed for mir5100 overexpressing cells. Summarizing, muscles that received miR425 transfected MIPCs regenerated more efficiently, as compared to muscles that received control cells.

## Discussion

SDF-1, known as CXCL12, is one of the crucial factors regulating cell migration. SDF-1 signaling is mediated by G-coupled protein receptors, i.e., CXCR4 and the atypical cytokine receptor CXCR7 that acts via β-arrestin not via G proteins [39]. In the present study, we noticed that the level of miR10a, miR151, miR425, miR5100 increased significantly when SC-derived myoblasts were treated with SDF-1 and decreased when *Cxcr4* expression was down-regulated in these cells. Therefore, we focused on these miRNAs to evaluate their role in stem and progenitor myogenic cell migration and differentiation. There is some evidence that miR10a, miR151, miR425, miR5100 could be involved in cell migration [40–43]. However, these molecules were mainly studied in cancer cells and little is known about their role under physiological conditions in tissue such as skeletal muscle.

miR10a, together with miR10b, is a member of the miR10 family. miR10 coding genes are present within the *Hox* gene cluster characterized by the presence of the DNA-binding homeobox domain and encoding proteins important in development and cancer [44–46]. miR10 was shown to directly regulate several transcripts of human *HOX* genes, such as *HOXA1, HOXA3,* or *HOXD10.* Furthermore, miR10a level changes in many types of cancer, such as glioblastoma, colon cancer, breast cancer, or acute myeloid leukemia [47–49]. miR10a promotes gastric cancer cell invasion through repression of *HOXD10* expression that results in up-regulation of *RHOC* expression and activation of the AKT signaling pathway [48]. miR10 also affected *KLF11* expression and increased TGFβR1 levels in human skin fibroblasts, what caused enhancement of cell migration [48]. miR151 is an important regulator of cell migration. It is encoded within FAK encoding gene [41, 50, 51]. In hepatocellular carcinoma cells, miR151 down-regulated the Rho GDP dissociation inhibitor α (RhoGDIA) coding transcript, and as a result RAC1, CDC42, and RHO were activated and cell migration was promoted [50]. Other studies showed that miR151 inhibited cell proliferation, tumor growth, and invasion by down-regulating the suppressor of cytokine signaling 5 (SOCS5) or TWIST1 in breast cancer cells, or leading to the suppression of PI3K/AKT phosphorylation in prostate cancer cells [51–53]. The overexpression of miR151 in breast cancer cells promoted proliferation and migration by leading to the inhibition of the phosphatase and tensin homolog (PTEN), a known tumor suppressor and negative regulator of PI3K/AKT [54]. Furthermore, miR425 was shown to promote gastric and bladder cancer progression, migration, and invasion through direct targeting of mRNA coding Dickkopf-related protein 3 (DKK3) [55, 56]. miR425 through down-regulation of SCAI or forkhead box D3 (FOXD3) promoted proliferation, migration, and hepatocellular carcinoma cell metastasis [57]. Next, miR5100 stimulated the proliferation and migration of oral squamous cell carcinoma cells by down-regulation of the suppressor of cancer cell invasion (SCAI) mRNA [43]. Interestingly, miR5100 was shown to support migration and epithelial-to-mesenchymal transition (EMT) of lung epithelial cells [58].

In the present study, analysis of the transcriptome of SC-derived myoblasts and MIPCs transfected with miRNA mimics showed that miR10a, miR151, miR425, and miR5100 were involved in RHO and actin signaling, that is, signaling engaged in cell migration [59, 60]. We confirmed that miR10a or miR425 overexpression led to drop of FAK and activated FAK levels, as well as β-actin, and that this reaction was observed mainly in MIPCs. Importantly, the changes were immediate, that is, observed within 48 h. Interestingly, cell migration was not affected by the majority of miRNAs tested, except miR10a, which increased SC-derived myoblast migration. Our previous studies documented SDF-1-induced migration of SC-derived myoblasts as well as other stem and stromal cells, such as mouse embryonic stem cells (ESCs) or BMSCs [25, 26, 61]. Furthermore, SDF-1 treatment altered MMP activity and actin organization via FAK, CDC42, and RAC-1 [23, 26]. Thus, we proposed that SDF-1 decreased miR10a, miR425, and miR5100 levels and through this mechanism prevented inhibition of FAK and actin expression.

Furthermore, we showed that miR10a, miR425, and miR5100 were involved in NOTCH signaling and changed the expression of NOTCH ligands, as well as *Hey1*, myogenic regulatory factors, and other muscle-specific proteins. The NOTCH signaling pathway is a very important regulator of SC activation and differentiation. The NOTCH receptor is activated by binding ligands such as DLL1, DLL4, JAG1, JAG2 or JAG4. Activation of NOTCH led to proteolysis of its intracellular domain, i.e., NICD, which is a transcriptional coactivator translocated to nucleus [62]. NICD interact with RBPJ to activate the expression of NOTCH-dependent genes such as *Hes*/*Hey* family [62]. Quiescent satellite cells express high levels of NOTCH1, 2, 3, and 4 and HEY1, HEYL, HES1 [62]. However, using C2C12 Beauchamp and coworkers showed that the expression of NOTCH1, NOTCH3, DLL1, DLL4 and JAG1 increased during myogenic differentiation [63]. After 5 days of differentiation, the presence of NOTCH1, DLL1, and DLL4 was limited to myotubes and the presence of *Notch3, Dll1,* and *Jag1* was characteristic for undifferentiated cells [63]. NOTCH activity sustained SCs in quiescent state [62, 64, 65]. Upon activation of SCs and during proliferation of myoblasts, NOTCH activity and *Hes/Hey* family expression decreases [62]. Constitutive activation of NOTCH, through NICD interaction with RBPJ and binding to two consensus sites of the gene, up-regulated PAX7 and promoted self-renewal of satellite cells [66]. Then, activation of NOTCH signaling in myocytes led to their dedifferentiation into PAX7-expressing satellite cells [67]. In addition, HES/HEY factors have been implicated in NOTCH-dependent repression of *Myod* [66, 68]. Ablation of NOTCH signaling resulted in up-regulation of MYOD, premature myogenic differentiation, and depletion of satellite cell pool [64, 69–72]. *Myod* expression is directly repressed by HES/HEY factors [68, 73]. Furthermore, MYOD enhanced and HES1 repressed *Dll1* expression in satellite cells. Down-regulation of HES1 increased and ablation of MYOD reduced *Dll1* expression level [73]. However, activation of NOTCH1 in myotubes was accompanied with DLL/JAG ligand expression [67]. In activated satellite cells, DLL1, HES1, and MYOD were up-regulated and their oscillatory level changed, but in differentiating myoblasts, HES1 was no longer expressed and the expression of *Dll1* and *Myod* was sustained [68, 73]. Similarly, studies focusing at mesoangioblasts, that is, the myogenic subset of muscle pericytes, showed that down-regulation of *Notch1* and *Dll1* resulted in NICD decrease [74]. However, NOTCH signaling activation by culture of cells in the presence of NOTCH ligands: DLL1, DLL4, or JAG1 promoted the expansion of human PAX7 + / MYOD- [75]. Treatment of cells with NOTCH ligands increased *Hes1, Hey1, HeyL* and *Pax7* and decreased *Myod* expression [75]. However, treatment with DLL1, DLL4 or JAG1 did not improve the engraftment ability of mouse and human SC-derived myoblasts [75]. Furthermore, treatment of mouse and human SC-derived myoblasts with DLL4 and PDGF-BB increased their self-renewal, trans-endothelial migration ability, expression of pericyte markers, and improved graft after intra-arterial transplantation to *Sgca-*null/scid/beige mice [76].

In our hands, the overexpression of miR10a, miR425, and miR5100 decreased the expression of *Dll1, Jag2.* miR425 and miR5100 overexpression resulted in the decrease in NICD level. Such phenotype was observed mainly in case of MIPCs. miR10a transfection led to *Hey1* expression increase in MIPC. Importantly, also the expression of *Pax7, Myf5, Myod, Mef2c*, *Myog*, *Musk* and *Myh3,* that is, the molecules involved in myogenesis, decreased in MIPCs transfected with miR10a, miR425, and miR5100. However, these changes did not affect myogenic differentiation in vitro nor in vivo*,* that is, after heterotopic transplantation. On the other hand, the miR5100 inhibitor increased myoblast fusion, miR10a decreased myoblast fusion, and miR425 surprisingly increased MIPC fusion. The influence of SDF-1 on myogenic differentiation is unclear. Odemis and coworkers reported that SDF-1 inhibited the differentiation of C2C12 and SC-derived myoblasts, as evaluated by MYOD, myosin heavy chain and/or myogenin expression [77]. On the other hand, Melchionna and coworkers showed that SDF-1 increased myogenic differentiation, as judged by myosin heavy chain accumulation and differentiation into myotubes by C2C12 and SC-derived myoblasts [78]. In our previous studies, we did not observe significant changes in SC-derived myoblast differentiation after SDF-1 treatment [26]. Currently observed changes in the transcriptome after miRNA mimic transfection did not improved significantly myogenic differentiation of analyzed cells. However, the role of miR10a, miR425, and miR5100 in the interaction of SDF-1 and NOTCH signaling in myogenic cells appears to be emerging. In particular, NOTCH signaling was shown to regulate CXCR4 expression in endothelial progenitor cells, endothelial cells, and bone marrow stromal cells [79].

Finally, transplantation of SC-derived myoblasts transfected with miR10a or control MIPCs decreased the number of new muscle fibers. On the other hand, transplantation of miR425 transfected MIPCs increased the number of new muscle fibers, as compared to control cells. Importantly, the level of MYOD, in skeletal muscles transplanted with miR425 transfected MIPCs, decreased what could correspond to lower number of myogenic precursor cells. Furthermore, in muscles transplanted with miR425 transfected MIPCs the level of MCK increased, corresponding to a higher number of new muscle fibers. These results are in agreement with the in vitro ability of miR425 transfected MIPCs and miR10a transfected SC-derived myoblasts to differentiate. However, the more efficient regeneration of muscles that received miR425 transfected MIPCs could be also a result of injected cells paracrine effect. Such impact was described for several cell types used to support skeletal muscle regeneration [80–83].

## Conclusions

**Fig. 9:**
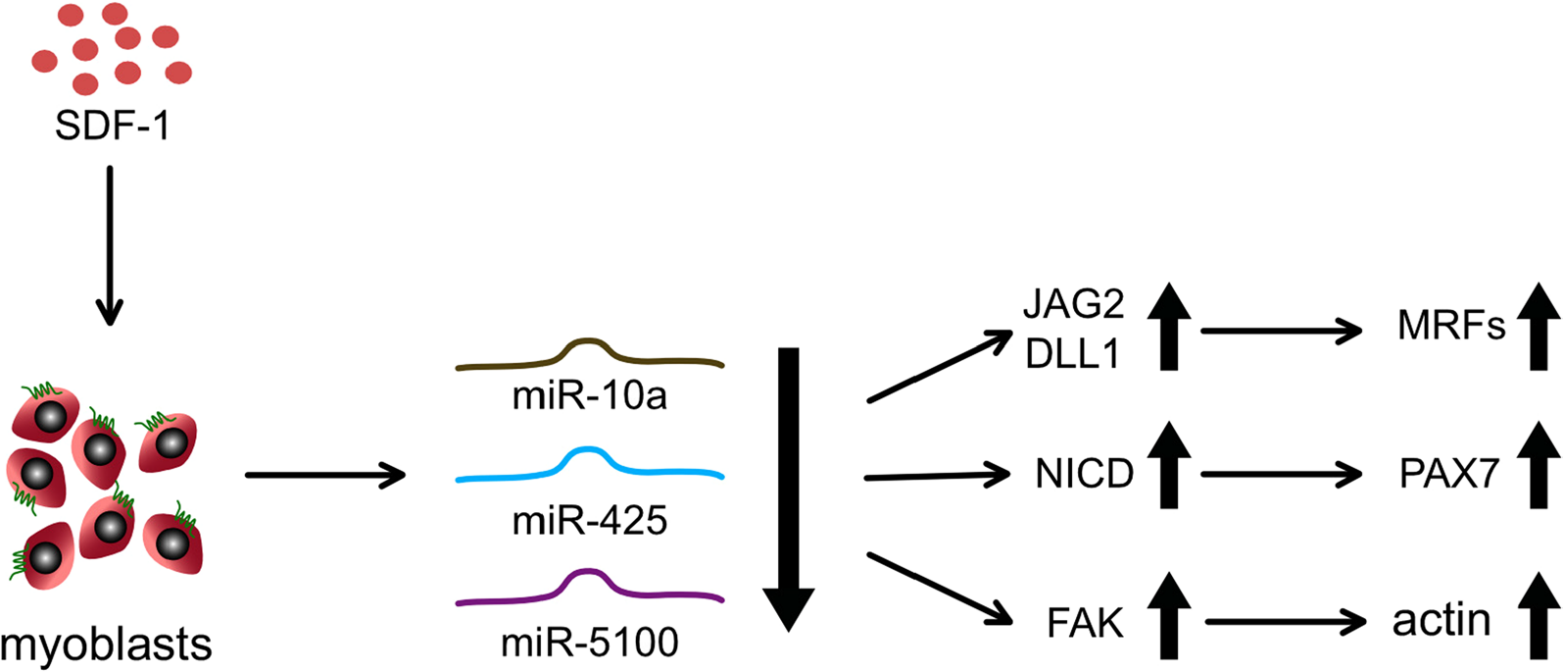
Suggested mechanism of SDF1 dependent miRNAs action. SDF1 dependent decrease in miR10a, miR425, and miR5100 allows the increase in the level of JAG2/DLL1, NICD, and FAK what results in the increase in the MRFs, PAX7, and actin levels.

We found that SDF-1 treatment of mouse myoblasts led to down-regulation of the miR10a, miR151, miR425, and miR5100 in CXCR4-dependent manner. miR10a, miR425, and miR5100 regulated the level of transcripts involved in NOTCH signaling, including *Dll1* and *Jag2*. Furthermore, miR10a, miR425, and miR5100 negatively regulated the transcripts involved in cell migration, as well as myogenic differentiation. We suggest that SDF-1 treatment down-regulates miR10a, miR425, and miR5100, affecting NOTCH signaling, migration, and differentiation of myogenic cells (Fig. 9). Moreover, MIPCs transfected with miR425 differentiated with higher efficiency in vitro*.* Furthermore, the muscles that received miR425 transfected MIPCs regenerated more efficiently, as compared to those injected with control cells.

### Supplementary Information

**Additional file 1. Fig. S1**. IPA analysis of changes in different cellular processes in SC-derived myoblasts and MIPCs transfected with miR10a, miR425 or miR5100 mimics or inhibitors. **Figure S2**. IPA analysis of changes in gene expression in SC-derived myoblasts and MIPCs transfected with miR10a, miR425 or miR5100 mimics or inhibitors compared to control, non-transfected cells. Red—up-regulated transcripts; green—down-regulated transcripts; gray—sum of up-regulated and down-regulated transcripts. **Figure S3**. Selected transcripts significantly changed in SC-derived myoblasts or MIPCs transfected either with miR10a, or miR425 or miR5100 mimics or mimics together with their inhibitors miRNA. Original results were shown in Fig. 3. (A) SC-derived myoblasts analyzed 48 h post transfection; (B) MIPCs analyzed 48 h post transfection; (C) SC-derived myoblasts analyzed 7 days post transfection; (D) MIPCs analyzed 7 days post transfection. **Figure S4**. Impact of miR151 mimic or inhibitor on migration and fusion of control or transfected SC-derived myoblasts and MIPCs. (A) Cell migration assessed 6 and 24 h after performing a scratch. (B) Fusion index. Three independent analyzes were performed. **Figure S5**. Full-length images of blots presented in Fig. 4. (A) NICD (~ 110 KDa); (B) FAK (~ 120 KDa); (C) pFAK (~ 120 KDa); (D) β-actin (~ 40 KDa); (E) MYOD (~ 45 KDa); and (F) α-tubulin (~ 50 KDa).Three independent biological analyzes were performed. **Figure S6**. Full-length images of blots presented in Fig. 8. (A) MYOD (~ 45 KDa); (B) MCK (~ 45 KDa) and (C) α-tubulin (~ 50 KDa) Three independent analyzes were performed.

## Data Availability

The datasets generated during and analyzed during the current study are available from the corresponding author on request. Department of Cytology, Faculty of Biology, University of Warsaw, Miecznikowa 1 St, 02–096 Warsaw, Poland. ArrayExpress accession number E-MTAB-12500 https://www.ebi.ac.uk/biostudies/arrayexpress/studies/E-MTAB-12500?query=E-MTAB-12500.

## Abbreviations

CFSE: Carboxyfluorescein succinimidyl ester
ctx: Cardiotoxin
CDC42: Cell division control protein 42
CEE: Chicken embryo extract
DKK3: Dickkopf related protein 3
DMEM: Dulbecco’s modified Eagle medium
ESCs: Embryonic stem cells
EMT: Epithelial-to-mesenchymal transition
FAK: Focal adhesion kinase
FGF: Fibroblast growth factor
FOXD3: Forkhead box D3
HGF: Hepatocyte growth factor
HS: Horse serum
IPA: Ingenuity Pathway Analysis
IGF-1: Insulin-like growth factor 1
ILK: Integrin-linked kinase
IL: Interleukin
Mmp12: Metalloproteinase 12
miRNAs: MicroRNAs
MIPCs: Muscle interstitial progenitor cells
Musk: Muscle-specific kinase
MRF: Myogenic regulatory factors
Myog: Myogenin
NMJs: Neuromuscular junctions
NICD: NOTCH intracellular domain
PDAC: Pancreatic ducal adenocarcinoma cell
PTEN: Phosphatase and tensin homolog
qRT-PCR: Quantitative real-time PCR analysis
RAC-1: Ras-related C3 Botulinum Toxin Substrate 1
SCs: Satellite cells
SDF-1: Stromal cell-derived factor 1
SCAI: Suppressor of cancer cell invasion
SOCS5: Suppressor of cytokine signaling 5
TAC: Transcriptome Analysis Console
TGF-β: Transforming growth factor β
VEGF: Vascular endothelial growth factor

## References

[1] Tedesco FS, Dellavalle A, Diaz-Manera J, Messina G, Cossu G (2010). Repairing skeletal muscle: regenerative potential of skeletal muscle stem cells. J Clin Invest.

[2] Hardy D, Besnard A, Latil M, Jouvion G, Briand D, Thepenier C (2016). Comparative study of injury models for studying muscle regeneration in mice. PLoS ONE.

[3] Fletcher JE, Hubert M, Wieland SJ, Gong QH, Jiang MS (1996). Similarities and differences in mechanisms of cardiotoxins, melittin and other myotoxins. Toxicon.

[4] Yang W, Hu P (2018). Skeletal muscle regeneration is modulated by inflammation. J Orthopaedic Transl.

[5] Ciemerych MA, Archacka K, Grabowska I, Przewozniak M (2011). Cell cycle regulation during proliferation and differentiation of mammalian muscle precursor cells. Results Probl Cell Differ.

[6] Tidball JG, Villalta SA (2010). Regulatory interactions between muscle and the immune system during muscle regeneration. Am J Physiol Regul Integr Comp Physiol.

[7] Dumont NA, Bentzinger CF, Sincennes MC, Rudnicki MA (2015). Satellite cells and skeletal muscle regeneration. Compr Physiol.

[8] Yin H, Price F, Rudnicki MA (2013). Satellite cells and the muscle stem cell niche. Physiol Rev.

[9] Forcina L, Cosentino M, Musaro A (2020). Mechanisms regulating muscle regeneration: insights into the interrelated and time-dependent phases of tissue healing. Cells.

[10] Bloch-Gallego E (2015). Mechanisms controlling neuromuscular junction stability. Cell Mol Life Sci.

[11] Forcina L, Miano C, Pelosi L, Musaro A (2019). An overview about the biology of skeletal muscle satellite cells. Curr Genomics.

[12] Relaix F, Bencze M, Borok MJ, Der Vartanian A, Gattazzo F, Mademtzoglou D (2021). Perspectives on skeletal muscle stem cells. Nat Commun.

[13] von Maltzahn J, Jones AE, Parks RJ, Rudnicki MA (2013). Pax7 is critical for the normal function of satellite cells in adult skeletal muscle. Proc Natl Acad Sci USA.

[14] Charge SB, Rudnicki MA (2004). Cellular and molecular regulation of muscle regeneration. Physiol Rev.

[15] Tedesco FS, Moyle LA, Perdiguero E (2017). Muscle interstitial cells: a brief field guide to non-satellite cell populations in skeletal muscle. Methods Mol Biol.

[16] Mierzejewski B, Archacka K, Grabowska I, Florkowska A, Ciemerych MA, Brzoska E (2020). Human and mouse skeletal muscle stem and progenitor cells in health and disease. Semin Cell Dev Biol.

[17] Sacchetti B (2019). Post-natal, "mesenchymal" stem cells: the assayable skeletal potency. J Stem Cells Regener Med.

[18] Persichini T, Funari A, Colasanti M, Sacchetti B (2017). Clonogenic, myogenic progenitors expressing MCAM/CD146 are incorporated as adventitial reticular cells in the microvascular compartment of human post-natal skeletal muscle. PLoS ONE.

[19] Sacchetti B, Funari A, Remoli C, Giannicola G, Kogler G, Liedtke S (2016). No identical "mesenchymal stem cells" at different times and sites: human committed progenitors of distinct origin and differentiation potential are incorporated as adventitial cells in microvessels. Stem Cell Rep.

[20] Dellavalle A, Sampaolesi M, Tonlorenzi R, Tagliafico E, Sacchetti B, Perani L (2007). Pericytes of human skeletal muscle are myogenic precursors distinct from satellite cells. Nat Cell Biol.

[21] Mierzejewski B, Grabowska I, Jackowski D, Irhashava A, Michalska Z, Streminska W (2020). Mouse CD146+ muscle interstitial progenitor cells differ from satellite cells and present myogenic potential. Stem Cell Res Ther.

[22] Kowalski K, Brzoska E, Ciemerych MA (2019). The role of CXC receptors signaling in early stages of mouse embryonic stem cell differentiation. Stem Cell Res.

[23] Kowalski K, Kolodziejczyk A, Sikorska M, Placzkiewicz J, Cichosz P, Kowalewska M (2017). Stem cells migration during skeletal muscle regeneration—the role of Sdf-1/Cxcr4 and Sdf-1/Cxcr7 axis. Cell Adh Migr.

[24] Kowalski K, Archacki R, Archacka K, Streminska W, Paciorek A, Golabek M (2016). Stromal derived factor-1 and granulocyte-colony stimulating factor treatment improves regeneration of Pax7-/- mice skeletal muscles. J Cachexia Sarcopenia Muscle.

[25] Brzoska E, Kowalski K, Markowska-Zagrajek A, Kowalewska M, Archacki R, Plaskota I (2015). Sdf-1 (CXCL12) induces CD9 expression in stem cells engaged in muscle regeneration. Stem Cell Res Ther.

[26] Brzoska E, Kowalewska M, Markowska-Zagrajek A, Kowalski K, Archacka K, Zimowska M (2012). Sdf-1 (CXCL12) improves skeletal muscle regeneration via the mobilisation of Cxcr4 and CD34 expressing cells. Biol Cell.

[27] O'Brien J, Hayder H, Zayed Y, Peng C (2018). Overview of microRNA biogenesis, mechanisms of actions, and circulation. Front Endocrinol.

[28] Gebert LFR, MacRae IJ (2019). Regulation of microRNA function in animals. Nat Rev Mol Cell Biol.

[29] Krol J, Loedige I, Filipowicz W (2010). The widespread regulation of microRNA biogenesis, function and decay. Nat Rev Genet.

[30] Potter ML, Smith K, Vyavahare S, Kumar S, Periyasamy-Thandavan S, Hamrick M (2021). Characterization of differentially expressed miRNAs by CXCL12/SDF-1 in human bone marrow stromal cells. Biomol Concepts.

[31] Jia D, Li Y, Han R, Wang K, Cai G, He C (2019). miR146a5p expression is upregulated by the CXCR4 antagonist TN14003 and attenuates SDF1induced cartilage degradation. Mol Med Rep.

[32] van Solingen C, de Boer HC, Bijkerk R, Monge M, van Oeveren-Rietdijk AM, Seghers L (2011). MicroRNA-126 modulates endothelial SDF-1 expression and mobilization of Sca-1(+)/Lin(-) progenitor cells in ischaemia. Cardiovasc Res.

[33] Periyasamy-Thandavan S, Burke J, Mendhe B, Kondrikova G, Kolhe R, Hunter M (2019). MicroRNA-141-3p negatively modulates SDF-1 expression in age-dependent pathophysiology of human and murine bone marrow stromal cells. J Gerontol A Biol Sci Med Sci.

[34] Fan Y, Xu LL, Shi CY, Wei W, Wang DS, Cai DF (2016). MicroRNA-454 regulates stromal cell derived factor-1 in the control of the growth of pancreatic ductal adenocarcinoma. Sci Rep.

[35] Rosenblatt JD, Lunt AI, Parry DJ, Partridge TA (1995). Culturing satellite cells from living single muscle fiber explants. In Vitro Cell Dev Biol Anim.

[36] Chen Y, Wang X (2020). miRDB: an online database for prediction of functional microRNA targets. Nucleic Acids Res.

[37] Agarwal V, Bell GW, Nam JW, Bartel DP (2015). Predicting effective microRNA target sites in mammalian mRNAs. Life.

[38] Kowalski K, Dos Santos M, Maire P, Ciemerych MA, Brzoska E (2018). Induction of bone marrow-derived cells myogenic identity by their interactions with the satellite cell niche. Stem Cell Res Ther.

[39] Gilbert W, Bragg R, Elmansi AM, McGee-Lawrence ME, Isales CM, Hamrick MW (2019). Stromal cell-derived factor-1 (CXCL12) and its role in bone and muscle biology. Cytokine.

[40] Espinosa Neira R, Salazar EP (2012). Native type IV collagen induces an epithelial to mesenchymal transition-like process in mammary epithelial cells MCF10A. Int J Biochem Cell Biol.

[41] Yue C, Chen X, Li J, Yang X, Li Y, Wen Y (2020). miR-151-3p inhibits proliferation and invasion of colon cancer cell by targeting close homolog of L1. J Biomed Nanotechnol.

[42] Zhou H, Liu W, Chen H, Ni J, Zhang Z, Xu A (2021). MicroRNA miR-425 inhibits proliferation and migration of colorectal cancer HCT116 cells. J Pak Med Assoc.

[43] Wei Z, Lyu B, Hou D, Liu X (2021). Mir-5100 mediates proliferation, migration and invasion of oral squamous cell carcinoma cells via targeting SCAI. J Investig Surg.

[44] Shah N, Sukumar S (2010). The Hox genes and their roles in oncogenesis. Nat Rev Cancer.

[45] Lund AH (2010). miR-10 in development and cancer. Cell Death Differ.

[46] Tehler D, Hoyland-Kroghsbo NM, Lund AH (2011). The miR-10 microRNA precursor family. RNA Biol.

[47] Zhi Y, Xie X, Wang R, Wang B, Gu W, Ling Y (2015). Serum level of miR-10-5p as a prognostic biomarker for acute myeloid leukemia. Int J Hematol.

[48] Liu F, Shi Y, Liu Z, Li Z, Xu W (2021). The emerging role of miR-10 family in gastric cancer. Cell Cycle.

[49] Liang T, Han L, Guo L (2020). Rewired functional regulatory networks among miRNA isoforms (isomiRs) from let-7 and miR-10 gene families in cancer. Comput Struct Biotechnol J.

[50] Ding J, Huang S, Wu S, Zhao Y, Liang L, Yan M (2010). Gain of miR-151 on chromosome 8q24.3 facilitates tumour cell migration and spreading through downregulating RhoGDIA. Nat Cell Biol.

[51] Yeh TC, Huang TT, Yeh TS, Chen YR, Hsu KW, Yin PH (2016). miR-151-3p targets TWIST1 to repress migration of human breast cancer cells. PLoS ONE.

[52] Liu C, Li W, Zhang L, Song C, Yu H (2019). Tumor-suppressor microRNA-151-5p regulates the growth, migration and invasion of human breast cancer cells by inhibiting SCOS5. Am J Transl Res.

[53] Chen S, Ke S, Cheng S, Wu T, Yang Y, Liao B (2020). MicroRNA-151 regulates the growth, chemosensitivity and metastasis of human prostate cancer cells by targeting PI3K/AKT. J BUON.

[54] Xiao S, Zhu H, Luo J, Wu Z, Xie M (2019). miR4255p is associated with poor prognosis in patients with breast cancer and promotes cancer cell progression by targeting PTEN. Oncol Rep.

[55] Ning JZ, Yu WM, Cheng F, Rao T, Ruan Y (2020). MiR-425 promotes migration and invasion in bladder cancer by targeting Dickkopf 3. J Cancer.

[56] Pei Y, Tang Z, Cai M, Yao Q, Xie B (2021). MicroRNA miR-425 promotes tumor progression by inhibiting Dickkopf-related protein-3 in gastric cancer. Bioengineered.

[57] Wu H, Shang J, Zhan W, Liu J, Ning H, Chen N (2019). miR4255p promotes cell proliferation, migration and invasion by directly targeting FOXD3 in hepatocellular carcinoma cells. Mol Med Rep.

[58] Li CY, Wang YH, Lin ZY, Yang LW, Gao SL, Liu T (2017). MiR-5100 targets TOB2 to drive epithelial-mesenchymal transition associated with activating smad2/3 in lung epithelial cells. Am J Transl Res.

[59] Nowakowski A, Walczak P, Lukomska B, Janowski M (2016). Genetic engineering of mesenchymal stem cells to induce their migration and survival. Stem Cells Int.

[60] Sadok A, Marshall CJ (2014). Rho GTPases: masters of cell migration. Small GTPases.

[61] Kowalski K, Archacki R, Archacka K, Stremińska W, Paciorek A, Gołąbek M, et al. Stromal derived factor-1 and granulocyte-colony stimulating factor treatment improves regeneration of Pax7−/− mice skeletal muscles. J Cachexia Sarcopenia Muscle. 2015. 10.1002/jcsm.12092 PMC4863826 27239402

[62] Mourikis P, Tajbakhsh S (2014). Distinct contextual roles for Notch signalling in skeletal muscle stem cells. BMC Dev Biol.

[63] Low S, Barnes JL, Zammit PS, Beauchamp JR (2018). Delta-like 4 activates notch 3 to regulate self-renewal in skeletal muscle stem cells. Stem Cells.

[64] Mourikis P, Sambasivan R, Castel D, Rocheteau P, Bizzarro V, Tajbakhsh S (2012). A critical requirement for notch signaling in maintenance of the quiescent skeletal muscle stem cell state. Stem Cells.

[65] Brack AS, Conboy IM, Conboy MJ, Shen J, Rando TA (2008). A temporal switch from notch to Wnt signaling in muscle stem cells is necessary for normal adult myogenesis. Cell Stem Cell.

[66] Wen Y, Bi P, Liu W, Asakura A, Keller C, Kuang S (2012). Constitutive Notch activation upregulates Pax7 and promotes the self-renewal of skeletal muscle satellite cells. Mol Cell Biol.

[67] Bi P, Yue F, Sato Y, Wirbisky S, Liu W, Shan T, et al. Stage-specific effects of Notch activation during skeletal myogenesis. eLife 2016;5. 10.7554/eLife.17355 PMC5070950 27644105

[68] Lahmann I, Brohl D, Zyrianova T, Isomura A, Czajkowski MT, Kapoor V (2019). Oscillations of MyoD and Hes1 proteins regulate the maintenance of activated muscle stem cells. Genes Dev.

[69] Bjornson CR, Cheung TH, Liu L, Tripathi PV, Steeper KM, Rando TA (2012). Notch signaling is necessary to maintain quiescence in adult muscle stem cells. Stem Cells.

[70] Mourikis P, Gopalakrishnan S, Sambasivan R, Tajbakhsh S (2012). Cell-autonomous Notch activity maintains the temporal specification potential of skeletal muscle stem cells. Development.

[71] Conboy IM, Conboy MJ, Smythe GM, Rando TA (2003). Notch-mediated restoration of regenerative potential to aged muscle. Science.

[72] Conboy IM, Rando TA (2002). The regulation of Notch signaling controls satellite cell activation and cell fate determination in postnatal myogenesis. Dev Cell.

[73] Zhang Y, Lahmann I, Baum K, Shimojo H, Mourikis P, Wolf J (2021). Oscillations of Delta-like1 regulate the balance between differentiation and maintenance of muscle stem cells. Nat Commun.

[74] Quattrocelli M, Costamagna D, Giacomazzi G, Camps J, Sampaolesi M (2014). Notch signaling regulates myogenic regenerative capacity of murine and human mesoangioblasts. Cell Death Dis.

[75] Sakai H, Fukuda S, Nakamura M, Uezumi A, Noguchi YT, Sato T (2017). Notch ligands regulate the muscle stem-like state ex vivo but are not sufficient for retaining regenerative capacity. PLoS ONE.

[76] Gerli MFM, Moyle LA, Benedetti S, Ferrari G, Ucuncu E, Ragazzi M (2019). Combined notch and PDGF signaling enhances migration and expression of stem cell markers while inducing perivascular cell features in muscle satellite cells. Stem Cell Rep.

[77] Odemis V, Boosmann K, Dieterlen MT, Engele J (2007). The chemokine SDF1 controls multiple steps of myogenesis through atypical PKCzeta. J Cell Sci.

[78] Melchionna R, Di Carlo A, De Mori R, Cappuzzello C, Barberi L, Musaro A (2010). Induction of myogenic differentiation by SDF-1 via CXCR4 and CXCR7 receptors. Muscle Nerve.

[79] Xie J, Wang W, Si JW, Miao XY, Li JC, Wang YC (2013). Notch signaling regulates CXCR4 expression and the migration of mesenchymal stem cells. Cell Immunol.

[80] Sassoli C, Pini A, Chellini F, Mazzanti B, Nistri S, Nosi D (2012). Bone marrow mesenchymal stromal cells stimulate skeletal myoblast proliferation through the paracrine release of VEGF. PLoS ONE.

[81] Archacka K, Grabowska I, Mierzejewski B, Graffstein J, Gorzynska A, Krawczyk M (2021). Hypoxia preconditioned bone marrow-derived mesenchymal stromal/stem cells enhance myoblast fusion and skeletal muscle regeneration. Stem Cell Res Ther.

[82] Wosczyna MN, Konishi CT, Perez Carbajal EE, Wang TT, Walsh RA, Gan Q, et al. Mesenchymal stromal cells are required for regeneration and homeostatic maintenance of skeletal muscle. Cell Rep. 2019;27(7):2029–35 e5. 10.1016/j.celrep.2019.04.074 PMC7034941 31091443

[83] Maeda Y, Yonemochi Y, Nakajyo Y, Hidaka H, Ikeda T, Ando Y (2017). CXCL12 and osteopontin from bone marrow-derived mesenchymal stromal cells improve muscle regeneration. Sci Rep.

