# Supplementary material for: SDF-1 and NOTCH signaling in myogenic cell differentiation: the role of miRNA10a, 425, and 5100
Source: Stem Cell Res Ther. 2023 Aug 15;14:204. doi: 10.1186/s13287-023-03429-x (PMC10426160; doi:10.1186/s13287-023-03429-x)

Figure S1

A

myoblasts

mimics

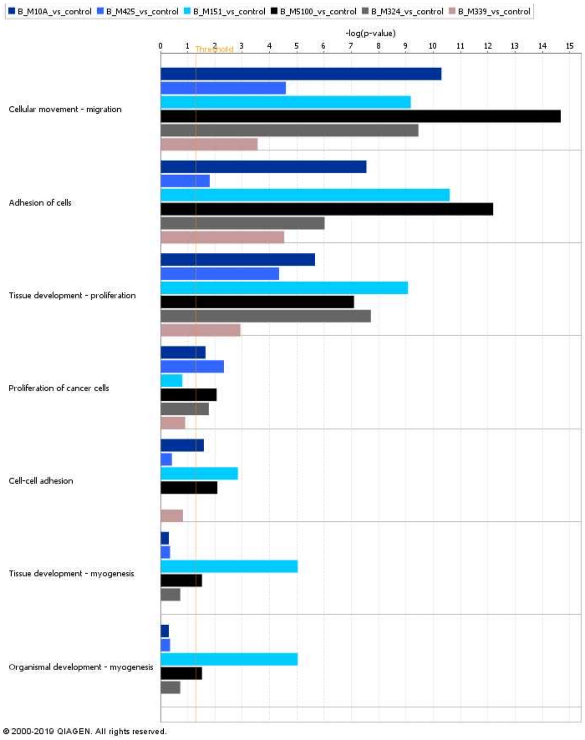

inhibitors

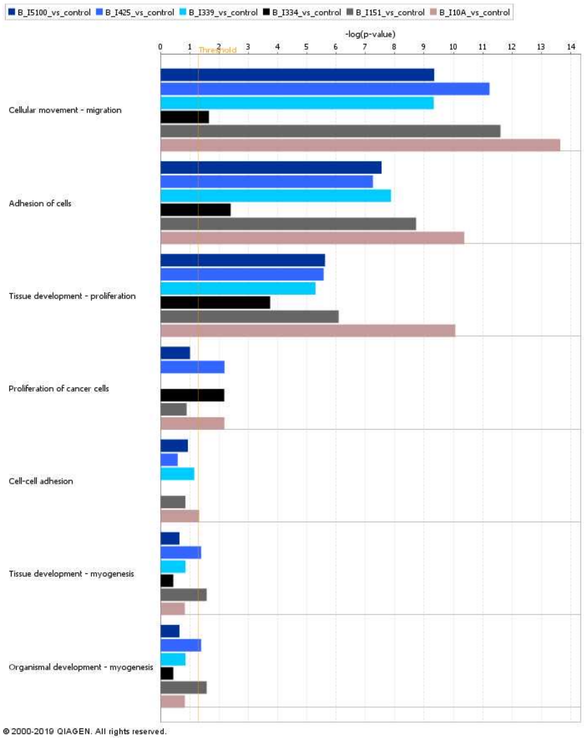

B

MIPCs

mimics

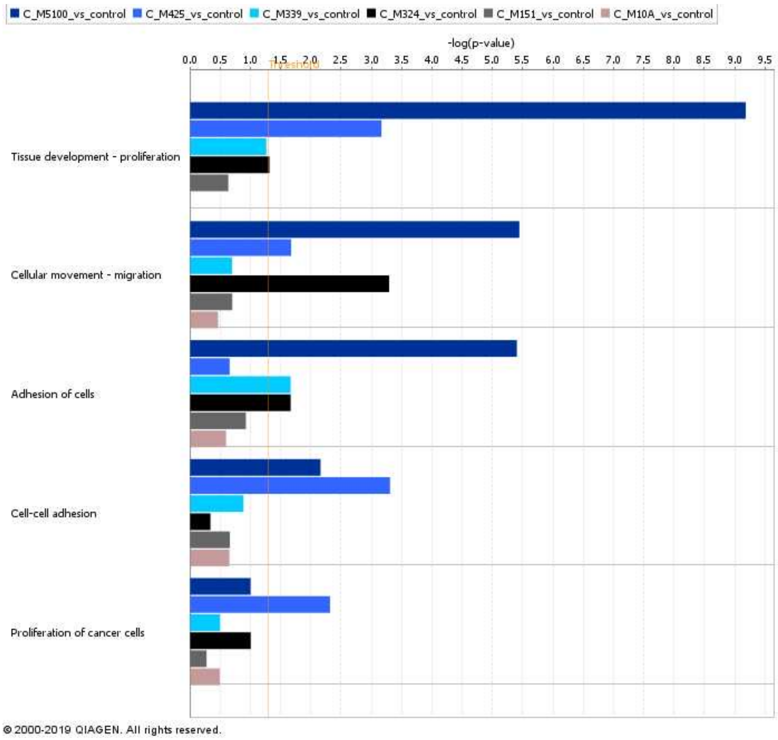

inhibitors

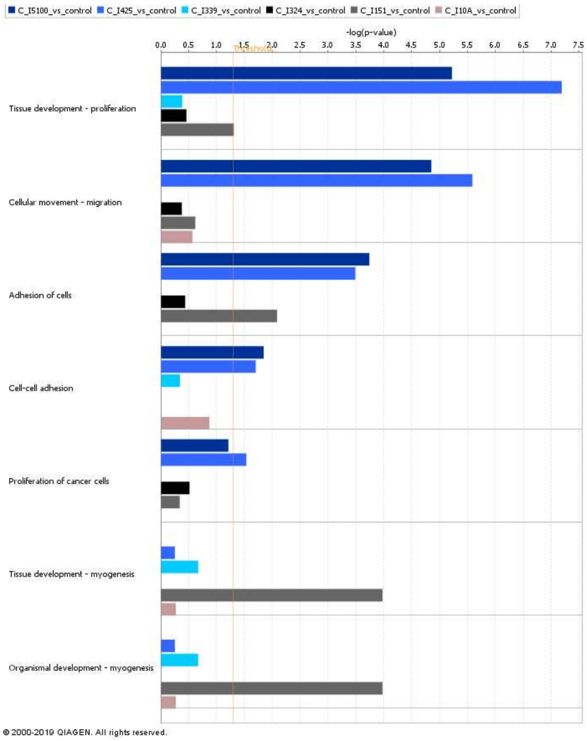

Figure S2

A

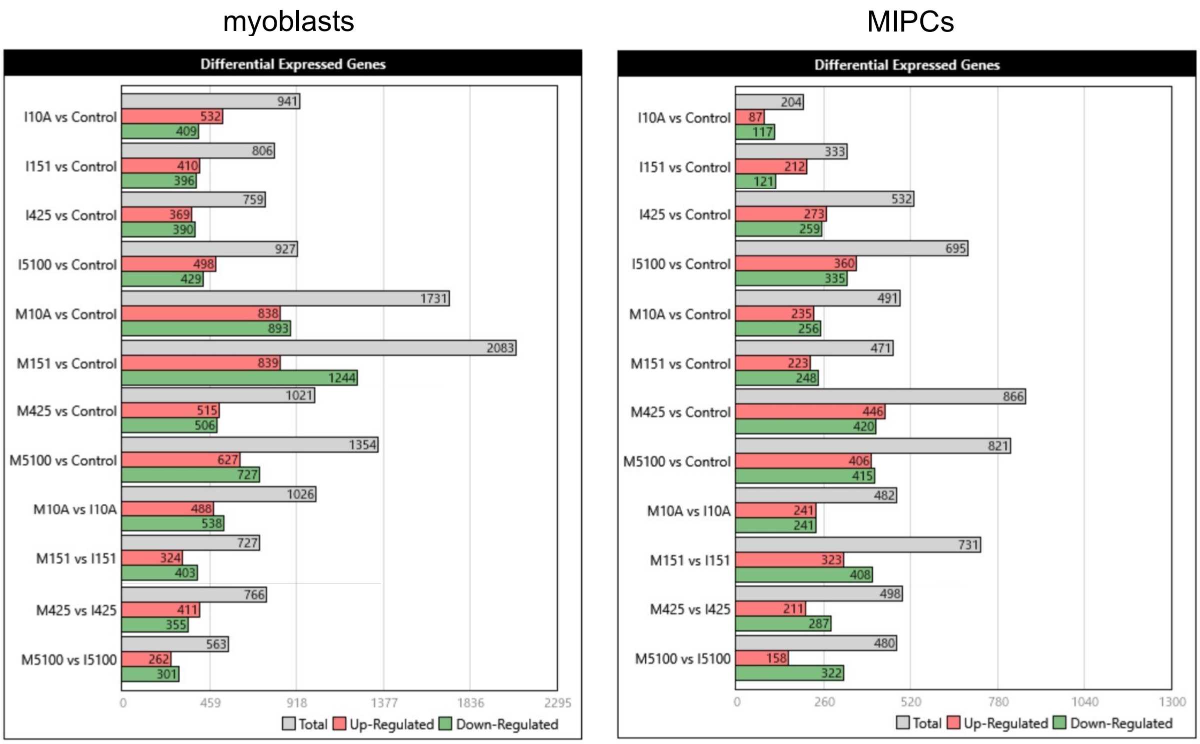

myoblasts
Differential Expressed Genes
I10A vs Control
941
532
409
I151 vs Control
806
410
396
I425 vs Control
759
369
390
I5100 vs Control
927
498
429
M10A vs Control
1731
838
893
M151 vs Control
2083
839
1244
M425 vs Control
1021
515
506
M5100 vs Control
1354
627
727
M10A vs I10A
1026
488
538
M151 vs I151
727
324
403
M425 vs I425
766
411
355
M5100 vs I5100
563
262
301
0
459
918
1377
1836
2295
Total
Up-Regulated
Down-Regulated
MIPCs
Differential Expressed Genes
I10A vs Control
204
87
117
I151 vs Control
333
212
121
I425 vs Control
532
273
259
I5100 vs Control
695
360
335
M10A vs Control
491
235
256
M151 vs Control
471
223
248
M425 vs Control
866
446
420
M5100 vs Control
821
406
415
M10A vs I10A
482
241
241
M151 vs I151
731
323
408
M425 vs I425
498
211
287
M5100 vs I5100
480
158
322
0
260
520
780
1040
1300
Total
Up-Regulated
Down-Regulated

Figure S3

A

	miR10a	miR10a/ inh10a	miR425	miR425/ inh425	miR5100	miR5100/ inh5100
Acta1						
Ilk				↓	↓	
Mmp12						
Dll1						
Jag1						
Jag2						
Hes1						
Hey1						
Pax7						
Myf5						
Myod		↑				
Mef2c						
Myog						
Musk						
Myh3						

B

	miR10a	miR10a/ inh10a	miR425	miR425/ inh425	miR5100	miR5100/ inh5100
Acta1	↓		↓		↓	
Ilk	↑			↓		↓
Mmp12	↓		↓	↓	↓	↓
Dll1	↓		↓		↓	
Jag1						
Jag2			↓	↑	↓	
Hes1						
Hey1	↑					
Pax7	↓		↓	↑	↓	
Myf5	↓		↓	↑	↓	↓
Myod	↓		↓		↓	
Mef2c	↓	↓	↓		↓	↓
Myog	↓		↓		↓	
Musk	↓		↓		↓	
Myh3	↓		↓		↓	

Figure S3

C

	miR10a	miR425	miR5100
Acta1			
Ilk			
Mmp12			
Dll1			
Jag1			
Jag2			
Hes1			
Hey1			
Pax7			
Myf5			
Myod			
Mef2c			
Myog			
Musk			
Myh3			

D

	miR10a	miR425	miR5100
Acta1			↓
Ilk			
Mmp12			
Dll1			
Jag1			
Jag2			
Hes1		↑	
Hey1			
Pax7			
Myf5		↓	↓
Myod			
Mef2c			↓
Myog			
Musk			
Myh3			

Figure S4

A

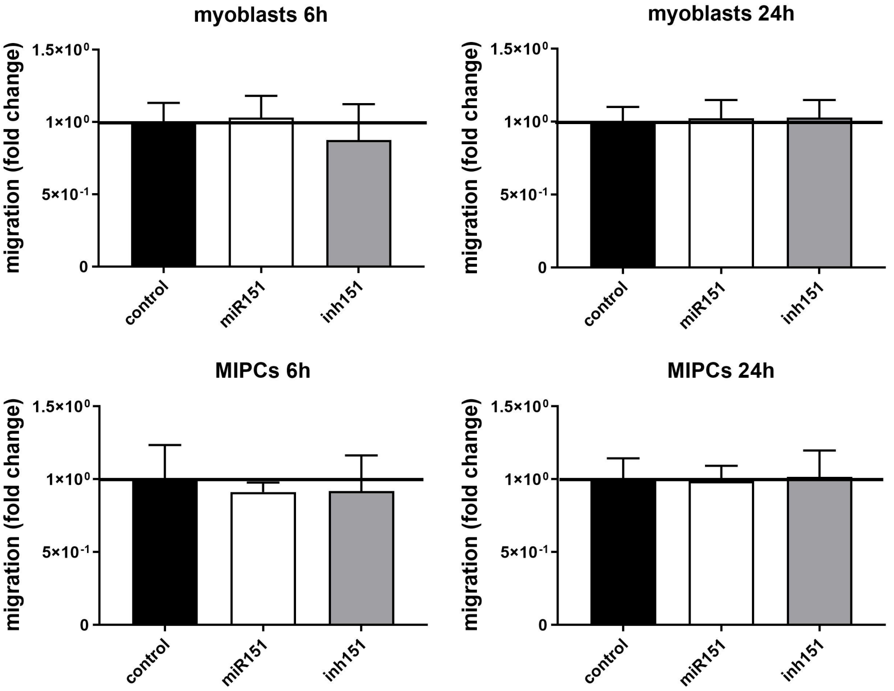
myoblasts 6h
myoblasts 24h
MIPCs 6h
MIPCs 24h
migration (fold change)
1.5×10⁰
1×10⁰
5×10⁻¹
0
control
miR151
inh151

B

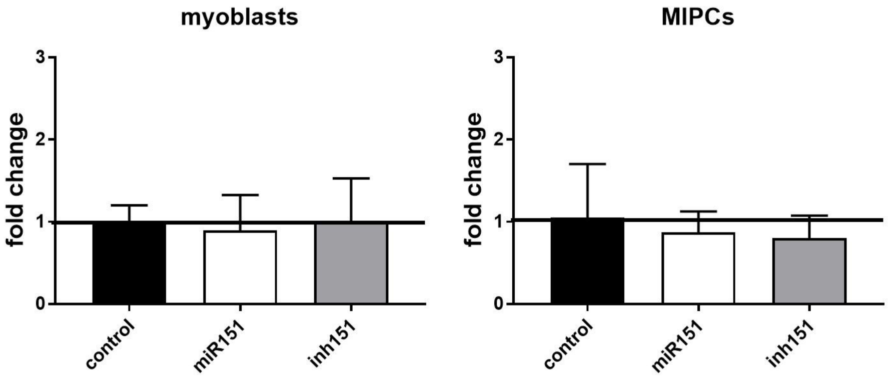
myoblasts
MIPCs
fold change
3
2
1
0
control
miR151
inh151

Figure S5

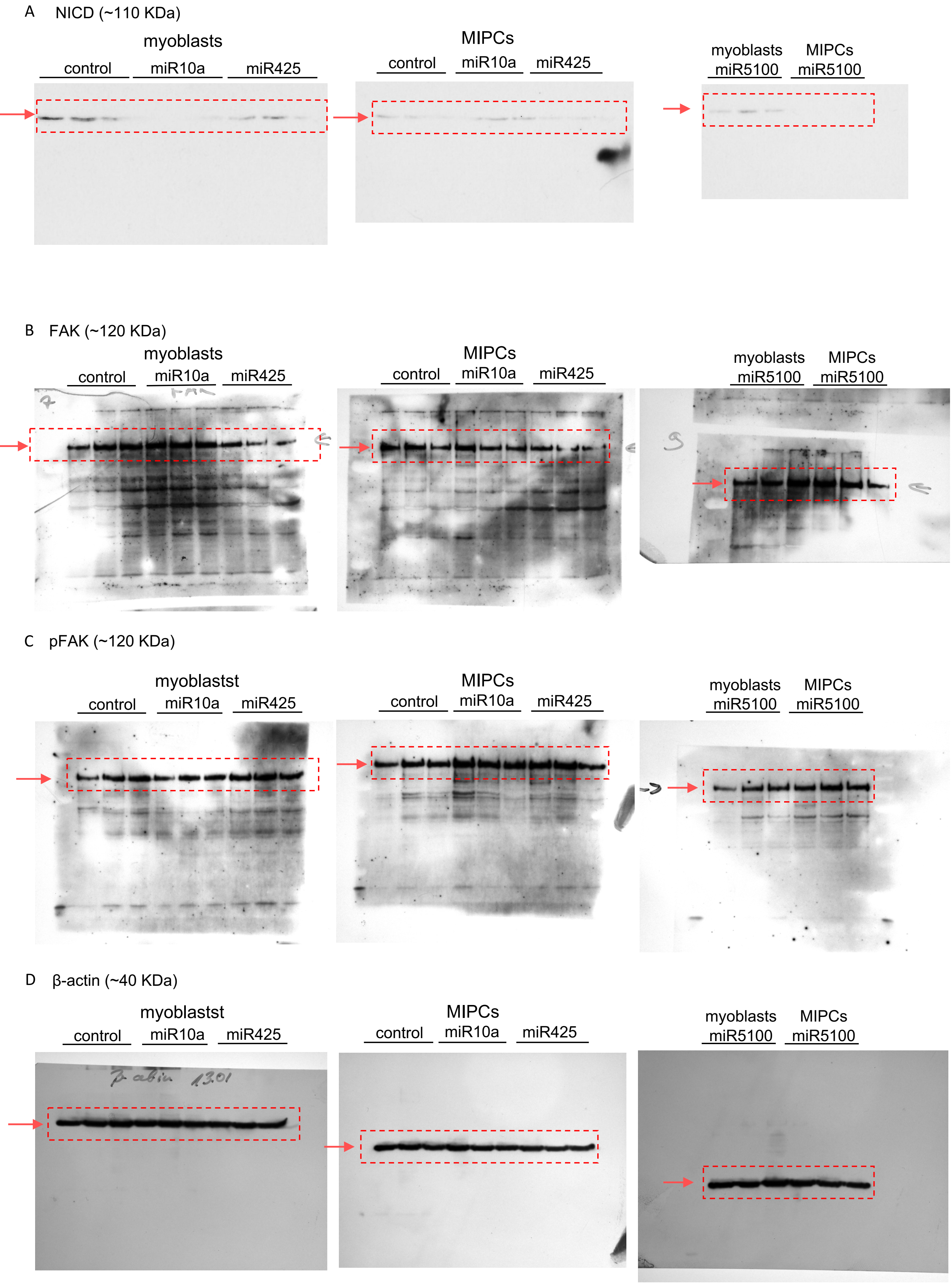
A NICD (~110 KDa)
myoblasts
control
miR10a
miR425
MIPCs
control
miR10a
miR425
myoblasts
miR5100
MIPCs
miR5100
B FAK (~120 KDa)
myoblasts
control
miR10a
miR425
MIPCs
control
miR10a
miR425
myoblasts
miR5100
MIPCs
miR5100
C pFAK (~120 KDa)
myoblastst
control
miR10a
miR425
MIPCs
control
miR10a
miR425
myoblasts
miR5100
MIPCs
miR5100
D β-actin (~40 KDa)
myoblastst
control
miR10a
miR425
MIPCs
control
miR10a
miR425
myoblasts
miR5100
MIPCs
miR5100

E MYOD (~45 KDa)

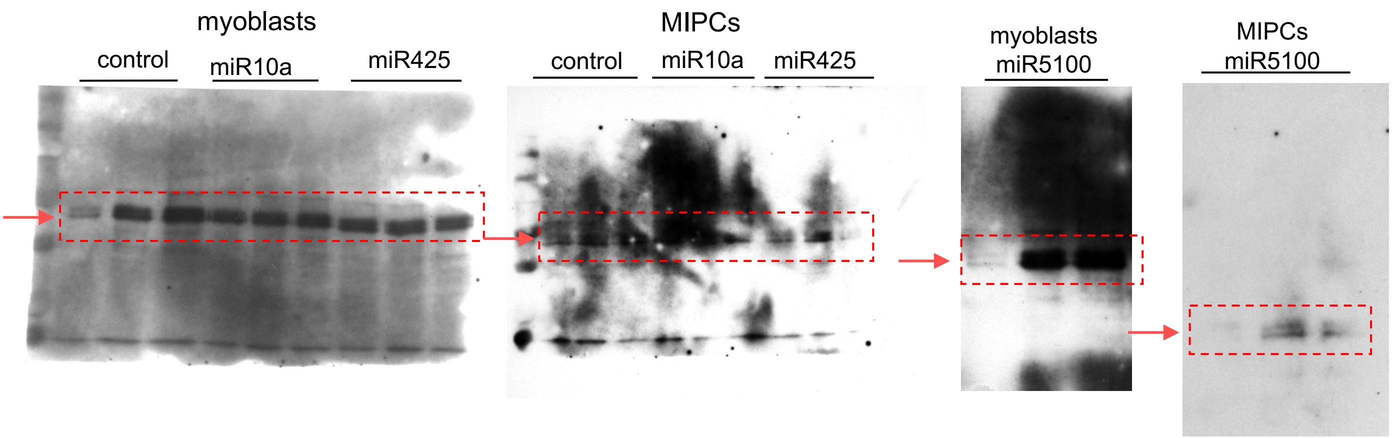

F α-tubulin (~50 KDa)

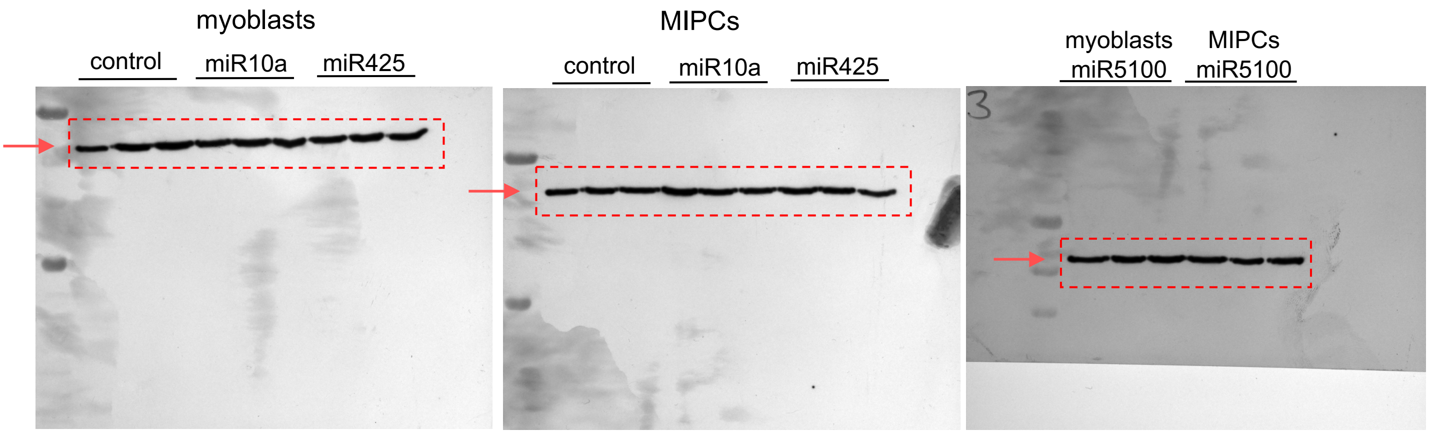

Figure S6

A MYOD (~45 KDa)

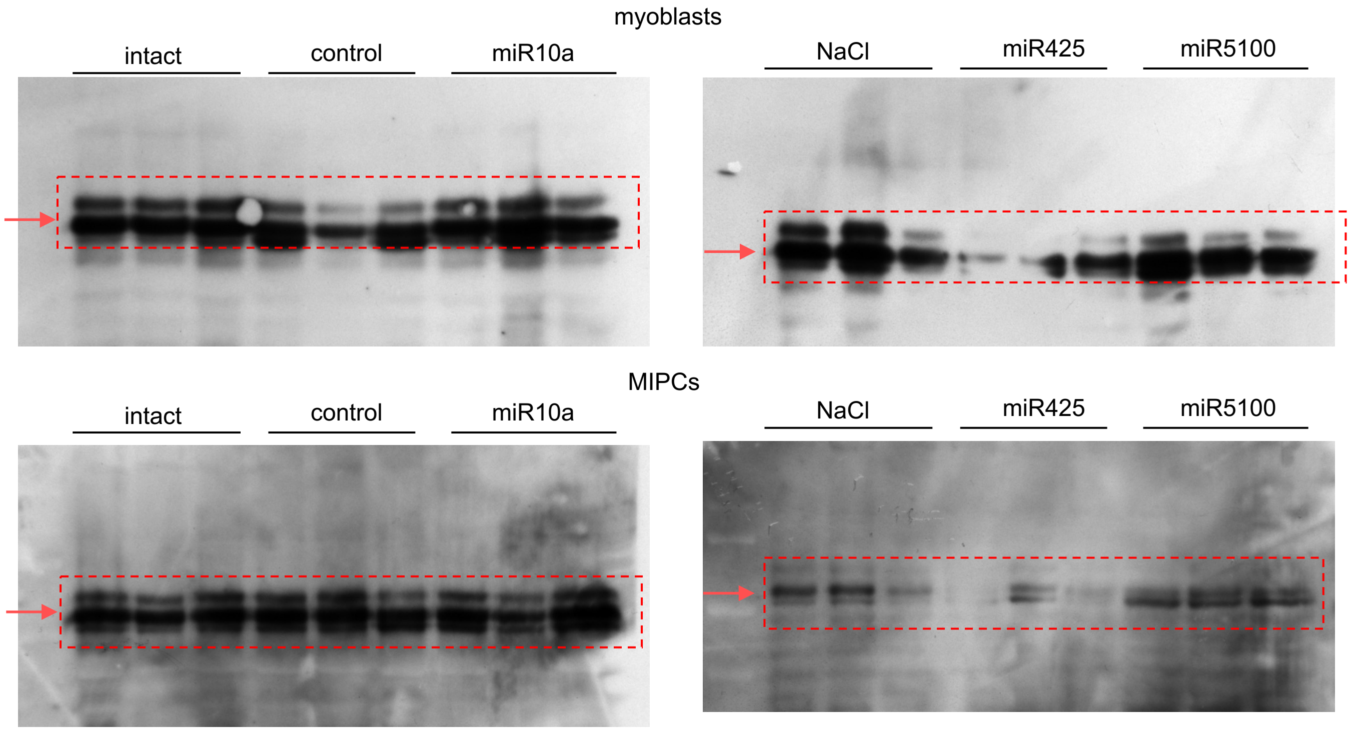

B MCK (~45 KDa)

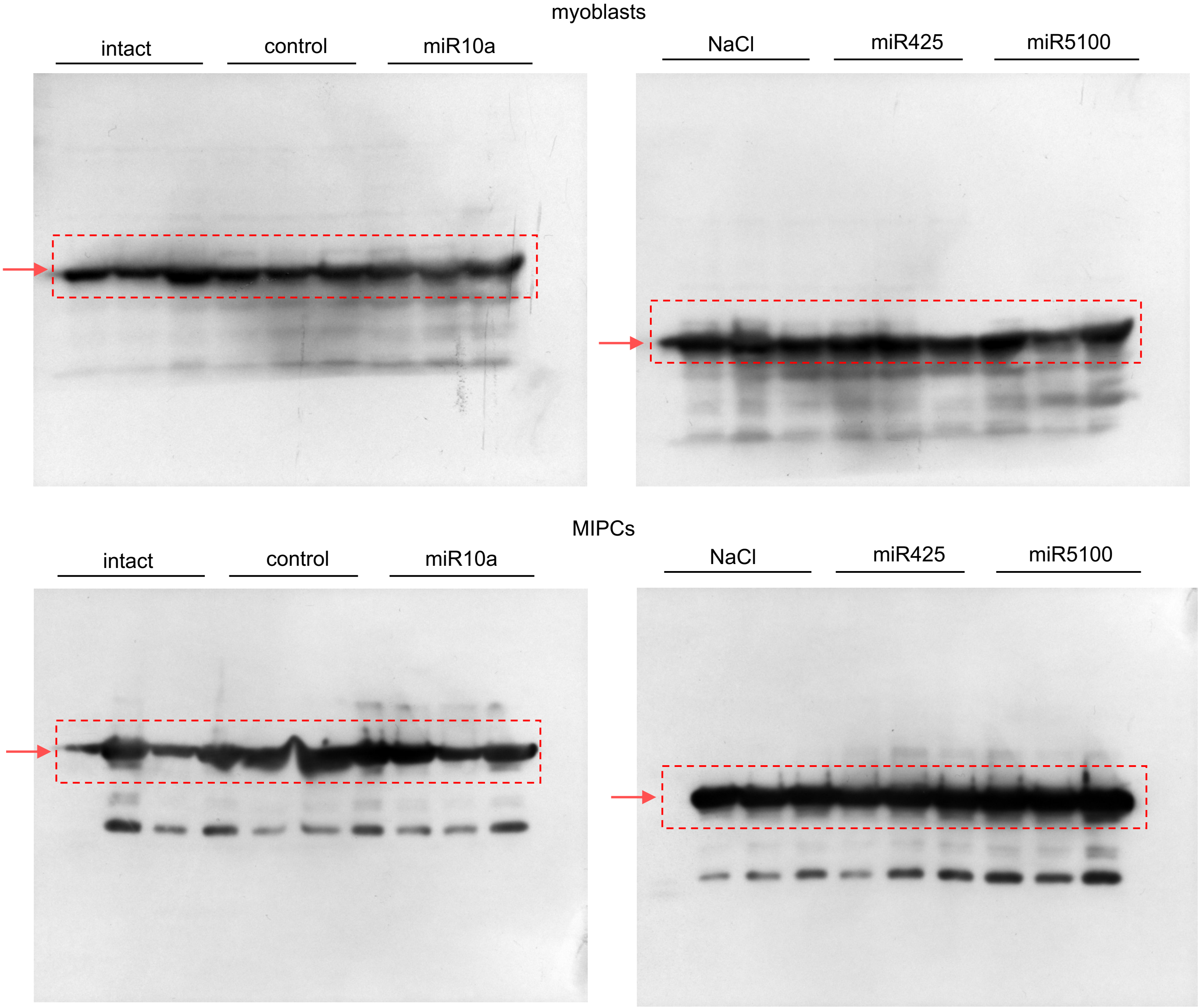

C α-tubulin (~50 KDa)

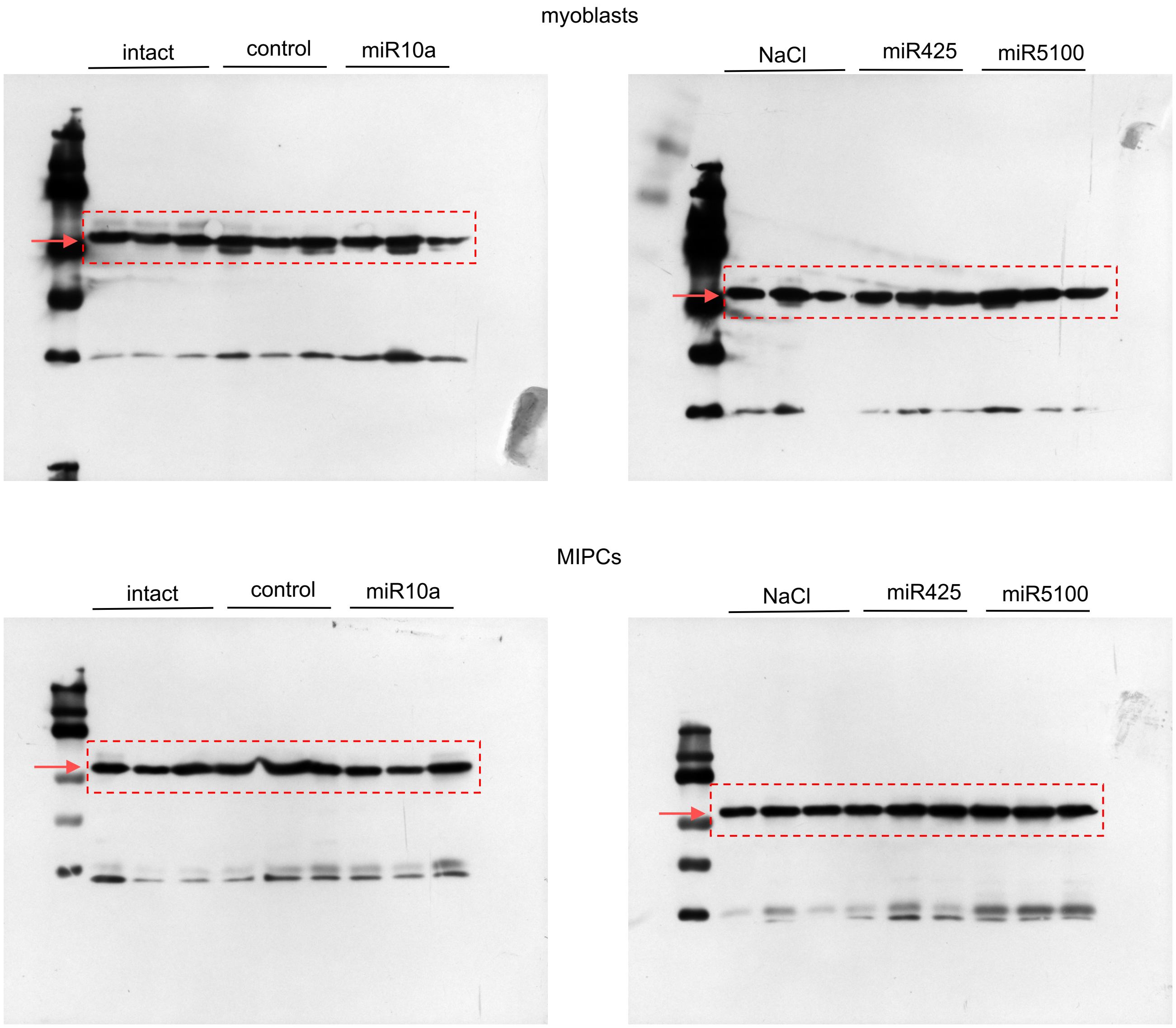

Supplement: Supplementary file 1 — Additional file 1. Fig. S1. IPA analysis of changes in different cellular processes in SC-derived myoblasts and MIPCs transfected with miR10a, miR425 or miR5100 mimics or inhibitors. Figure S2. IPA analysis of changes in gene expression in SC-derived myoblasts and MIPCs transfected with miR10a, miR425 or miR5100 mimics or inhibitors compared to control, non-transfected cells. Red—up-regulated transcripts; green—down-regulated transcripts; gray—sum of up-regulated and down-regulated transcripts. Figure S3. Selected transcripts significantly changed in SC-derived myoblasts or MIPCs transfected either with miR10a, or miR425 or miR5100 mimics or mimics together with their inhibitors miRNA. Original results were shown in Fig. 3. (A) SC-derived myoblasts analyzed 48 h post transfection; (B) MIPCs analyzed 48 h post transfection; (C) SC-derived myoblasts analyzed 7 days post transfection; (D) MIPCs analyzed 7 days post transfection. Figure S4. Impact of miR151 mimic or inhibitor on migration and fusion of control or transfected SC-derived myoblasts and MIPCs. (A) Cell migration assessed 6 and 24 h after performing a scratch. (B) Fusion index. Three independent analyzes were performed. Figure S5. Full-length images of blots presented in Fig. 4. (A) NICD (~ 110 KDa); (B) FAK (~ 120 KDa); (C) pFAK (~ 120 KDa); (D) β-actin (~ 40 KDa); (E) MYOD (~ 45 KDa); and (F) α-tubulin (~ 50 KDa).Three independent biological analyzes were performed. Figure S6. Full-length images of blots presented in Fig. 8. (A) MYOD (~ 45 KDa); (B) MCK (~ 45 KDa) and (C) α-tubulin (~ 50 KDa) Three independent analyzes were performed. [file 13287_2023_3429_MOESM1_ESM.pdf]
